# Untangling altruism and parochialism in human intergroup conflict

**DOI:** 10.1016/j.isci.2025.113978

**Published:** 2025-11-10

**Authors:** Robert Böhm, Luke Glowacki, Hannes Rusch, Isabel Thielmann

**Affiliations:** 1 Faculty of Psychology, University of Vienna, Universitatsstrase 7, 1010 Vienna, Austria; 2 Department of Banking and Finance, University of Innsbruck, Universitatsstrase 15, 6020 Innsbruck, Austria; 3 Department of Anthropology, Boston University, 232 Bay State Road, Suite 101, Boston, MA 02215, USA; 4 Max Planck Institute for the Study of Crime, Security and Law, Gunterstalstrase 73, 79100 Freiburg im Breisgau, Germany; 5 Department of Microeconomics and Public Economics, Maastricht University, P.O. Box 616, 6200 MD Maastricht, the Netherlands

**Keywords:** social sciences, psychology, research methodology, sociology

## Abstract

Human intergroup conflict occurs on a scale unmatched in other mammals. Paradoxically, this capacity for war is closely linked to our exceptionally cooperative abilities. Models of “parochial altruism” describe how within-group cooperation and between-group competition may co-evolve, but it is unclear whether these models reflect human preference adaptation in real-world conflicts. Across five studies (total *N* = 1,121), we develop and validate a psychometric toolkit to test the core assumptions and predictions of parochial altruism models in groups involved in real conflicts of varying intensities. Our measures clearly distinguish interindividual altruism from intergroup parochialism, outperform prior metrics in capturing social preferences related to intergroup conflict, and improve predictions of individuals’ conflict contributions. Notably, we find that parochialism varies for different outgroups—an unanticipated result that challenges existing theoretical models. Our work provides new tools for studying individual- and group-level social preferences in intergroup relations and presents novel evidence to inform substantive theoretical improvement.

## Highlights

• We develop and validate a toolkit for measuring preferences in real-world conflicts
• Our toolkit distinguishes interindividual altruism from intergroup parochialism
• Separating these two preferences improves predictions of conflict behavior
• Parochialism varied for different outgroups, challenging theories of parochial altruism

## Graphical abstract

**Figure:**
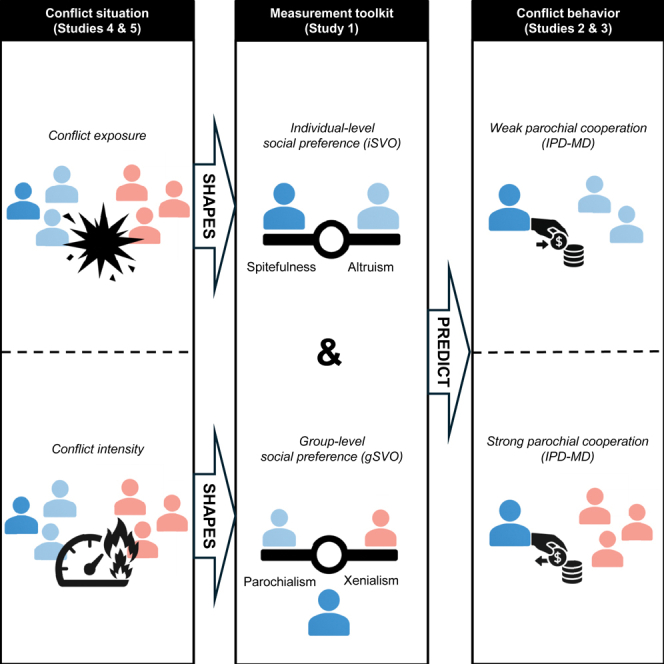

## Introduction

Human history abounds with intergroup violence and war.^1^^,^^2^^,^^3^^,^^4^ Such hostilities between groups are created by, and attractive for, individuals with extreme levels of certain dispositional tendencies.^5^^,^^6^^,^^7^^,^^8^ However, conflicts and the collateral damages they cause shape the mentality of entire populations. The resulting expanding spirals of hatred, violence, counter-hatred, and counter-violence can be considered “psychological war traps.”^4^^,^^9^^,^^10^^,^^11^

Over the last two decades, a range of influential models were developed to describe the dynamics that may unfold in populations composed of agents who are able to exhibit different behaviors toward in- and out-group members.^3^^,^^12^^,^^13^ Most widely known among these models is probably the one by Choi and Bowles.^14^ Their parochial altruism model assumes that agents’ behavior is determined by the combination of two independent *preferences*. The first preference describes how an individual values their own outcomes relative to those of ingroup members and distinguishes *altruistic* individuals, who contribute to producing public goods for the ingroup at a personal cost, from non-altruistic individuals. The second preference concerns the comparison of ingroup to outgroup members’ welfare and distinguishes *parochial* individuals, who avoid peaceful interactions with outgroups and provoke costly intergroup conflicts, from tolerant individuals. Over longer time horizons, then, the model predicts evolving populations to oscillate between two states: relatively peaceful times, during which tolerant non-altruists are most prevalent, and warlike times, during which parochial altruists are more prevalent. While many details of Choi and Bowles^14^ are debatable, including their very definition of “parochialism,” their main contribution can arguably be seen in reviving and fleshing out Darwin’s idea on how proclivities for within-group prosociality and between-group hostility can co-evolve dynamically.^13^

Importantly, most models in the fashion of the seminal model by Choi and Bowles^14^ can be interpreted in two ways, which have developed distinct lives in the literature since.^15^ For one, there is the “biological” interpretation of such models as describing populations’ underlying genetic makeup and its Darwinian evolution over longer time frames. Choi and Bowles^14^ emphasized this interpretation and proposed several auxiliary assumptions to calibrate their model to this context.^14^^,^^16^ These auxiliary assumptions were heavily criticized later; though, to the effect that most scholars consider the biological interpretation of the model implausible today.^3^^,^^12^^,^^13^^,^^17^^,^^18^ However, unaffected by these limitations of the biological interpretation, the alternative interpretation of such models views them as describing the more fast-paced dynamics of *preference* change during individual agents’ lifetimes or across a few generations. Whether this “cultural” interpretation better applies to, and predicts, the fundamental dynamics of preference adaptation in the context of war and peace remains to be scrutinized. This is what we aim at in the present paper.

We study whether two core assumptions of preference-based parochial altruism models apply to human decision-making in situations of intergroup conflict. We consider testing these two assumptions crucial for deciding whether further research on parochial altruism as a “cultural adaptation” promises meaningful results. Moreover, we test two directional predictions of preference change made by Choi and Bowles.^14^ Although our studies are not designed to determine the causal directionality of preference change, our results can nonetheless shed light on the psychological mechanisms underlying preference dynamics in the context of conflict—an important task, since the proximate workings of these mechanisms are largely left in the dark by previous theories. Thus, the measures we develop and (mixed) results on preference dynamics we report in the present paper are informative for all theories that conceptualize “parochial altruism” as a nexus of two co-evolving behavioral preferences.

### Related literature and research design

Thorough scrutiny of the cultural interpretation of any parochial altruism model requires testing two key assumptions (which we label as “A1” and “A2”). Moreover, the model by Choi and Bowles^14^ makes two testable predictions about the direction of the co-evolution between altruism and parochialism (labeled as “P1” and “P2”). This section introduces them, relates them to the previous literature, and explains our research design.

#### Assumption A1: “Altruism and parochialism are separate social preferences.”

First, any preference-based parochial altruism model assumes that individuals’ social preferences can be meaningfully decomposed along two dimensions: (individual-level) altruism/non-altruism and (group-level) parochialism/tolerance. Previous research has shown that (individual-level) altruistic *preferences* and both altruistic and parochial *behaviors* vary substantially across individuals and populations.^19^^,^^20^^,^^21^^,^^22^ However, this earlier research missed out on assessing (group-level) parochial preferences *independently* from (individual-level) altruistic preferences. Earlier studies only indirectly inferred group-level preferences from multiple measurements of individual-level altruism toward recipients with different group memberships.^23^^,^^24^^,^^25^ However, this approach is tainted by the methodologically induced correlation between the altruism measures used as inputs. A critical example is fully selfish individuals: in trading off outcomes for self vs. outcomes for others, purely selfish individuals will always maximize their own payoff irrespective of others’ group membership, thereby reducing others’ outcomes to a minimum. When such minimized outcomes for others are used to infer group-level preferences, these inferred preferences are distorted toward non-parochialism and may, in the extreme case where all outcomes for others are zero, even remain unobservable. Removing this limitation, and to allow for a conclusive empirical test of assumption A1, we devise a novel measurement toolkit, consisting of *conceptually orthogonal* measures of individual-level and group-level social preferences. We validate the measures’ psychometric properties in a convenience sample in Study 1. All subsequent studies then present consecutive tests of assumption A1: if individual- and group-level social preferences are independent, we should expect, at most, moderate correlations between them when measured repeatedly across different samples.

#### Assumption A2: “Altruism and parochialism independently contribute to predicting intergroup aggression.”

Second, preference-based parochial altruism models assume that altruistic and parochial preferences within individual agents each contribute to predicting harmful *behavior* toward outgroups in intergroup conflict settings. A handful of studies using economic games supposed to model (certain aspects of) intergroup conflict tested the predictive power of (individual-level) altruistic preferences for ingroup beneficial *behavior* that imposes costs on outgroups.^26^^,^^27^^,^^28^ Critically, however, these studies are silent on the independent role of (group-level) parochial preferences. Therefore, in Studies 2 and 3, we test assumption A2 by eliciting altruistic and parochial preferences from members of natural groups with high rivalry and investigating whether both contribute to explaining behavior in economic games that allow participants to inflict (financial) damage on outgroup members. We consider A2 to be violated if the inclusion of one preference dimension (and possibly its interaction with the other) fails to improve behavioral predictions beyond those based solely on the other.

#### Predictions P1 and P2: “Exposure to intergroup conflict affects individuals’ preferences, such that they become more altruistic (P1) and more parochial (P2).”

In addition to these assumptions, which all preference-based parochial altruism models need to make, the model by Choi and Bowles^14^ predicts *preference change* during longer phases of conflict (vs. peace), such that individuals become more (vs. less) parochial and more (vs. less) altruistic over time. Note that P1 and P2 also translate into predictions about the prevalence of different *preference types* at the (local) population level—i.e., we should observe more parochial altruists in populations that are directly affected by intergroup conflicts relative to populations in more peaceful environments.

Consistent with P1, it is quite robustly established that exposure to intergroup conflict increases individuals’ prosocial *behavior* toward ingroup members.^29^^,^^30^^,^^31^ However, whether conflict exposure also influences individuals’ individual- and group-level *preferences*, i.e., increases altruism and parochialism, is much less clear.^32^^,^^33^^,^^34^ To close this gap, we provide quasi-experimental tests of predictions P1 and P2. Specifically, in Study 4, we examine altruistic and parochial preferences in the field among participants with high vs. low recent exposure to real-world violent intergroup conflict with a fixed outgroup. We then follow up with a preregistered quasi-experiment in Study 5, recruiting a US-sample to examine how the intensity of conflict that participants perceive for different pairings of in- and out-groups affects their altruistic and parochial preferences.

### Measuring individual- and group-level social preferences

The conceptual complexity resulting from the combination of individual- and group-level social preferences in addition to a somewhat lax treatment of the distinction between (revealed) *preferences* and (observed) *behavior* has produced considerable terminological confusion in the literature.^12^ Therefore, we start with a simple formalization to sharpen our terminological framework.

Assume an agent’s preferences to be described by the utility function

(Equation 1) $$u\left(x,x_{i},x_{o}\right)=x+\alpha \cdot x_{i}+\gamma \cdot x_{o}\text{,}$$

wherein *x* is the agent’s own payoff, *x*_*i*_ is a representative ingroup member’s payoff, *x*_*o*_ is a representative outgroup member’s payoff, and *α*,*γ*∈[-*∞*,*∞*] are the individual’s preference parameters. Think of this agent’s *individual-level* social preferences as being captured in *α*—just as in the formalization of social value orientation (SVO^35^). Accordingly, an agent’s individual-level social preference can be positive (*α* > 0, commonly referred to as “prosocial” or “altruistic”), nil (*α* = 0, “individualistic” or “selfish”), or negative (*α* < 0, “spiteful” or “competitive”). For all decisions which do not affect marked outgroup members, only *α* matters, as in these cases *x*_*o*_ = 0. If *x*_*o*_ ≠ 0, however, the agent’s *group-level* social preferences become relevant. Think of these as being captured in *γ* and label them “ingroup favoring” for *γ* < *α*, “universalist” if *γ* = *α*, and “outgroup favoring” if *γ* > *α*. Note that this is a definition of *γ* relative to *α*. Beyond this, the sign of *γ* captures additional information: for *γ* < 0 we have “outgroup hate,” *γ* = 0 “outgroup neglect,” and *γ* > 0 “outgroup love.”

In building our new measurement toolkit for separately assessing individual- and group-level social preferences, we capitalize on one of the most established measures of individual-level social preferences: the SVO slider measure.^35^^,^^36^ In its traditional form, the SVO slider measure is a one-dimensional measure of *α*. We label this dimension “iSVO” here. The iSVO measure is composed of six items, in which individuals make dictator game-like allocations affecting their own, think *x* in Equation 1, and an unknown ingroup member’s payoff, think *x*_*i*_. As the second dimension in our toolkit, we add a measure of group-level social preferences, “gSVO,” capturing the sign of *γ* and its relation to *α*—i.e., gSVO could be expressed as a coefficient *γ* = *g*/*α* in Equation 1, wherein *g* is inferred from participants’ choice behavior using a second set of dictator games. Importantly, gSVO is elicited by using the same dictator games as in the iSVO measure, but fixing *x* = 0 by asking participants to make their six allocation decisions for a marked ingroup member, think *x*_*i*_, and a marked outgroup member, think *x*_*o*_.

Thus, by taking individual-level self-interest, i.e., *x*, out of the elicitation procedure for group-level preferences and asking participants to trade off *x*_*i*_ directly against *x*_*o*_, we solve a critical measurement problem present in earlier work. However, note that to keep the elicitation procedure succinct, we chose not to measure how individuals trade off *x* against *x*_*o*_—i.e., we did not include dictator games involving “self” and an outgroup member. Theoretically, such a third set of dictator games also does not add more information as long as individuals have transitive preferences, which is often the case.^37^ Moreover, note that the simple linear utility framework of Equation 1 that we use for conceptual clarity formally breaks down for the extreme case when exactly *α* = 0. However, this is merely an expositional issue: all that is required for our measurement toolkit to produce meaningful results is that participants have complete preferences—i.e., participants should be able to decide whether they weakly prefer allocation (*x*,*x*_*i*_,*x*_*o*_) over $\left(x^{\prime },x_{i}^{\prime },x_{o}^{\prime }\right)$ or not for all possible combinations of allocations. Note that, as with all measures based on revealed preferences, individuals who are exactly indifferent between multiple options are expected to make random choices, which cannot be distinguished from irrational choices. These individuals thus add random noise to measures of sample characteristics, but should not introduce systematic biases.

**Figure 1:**
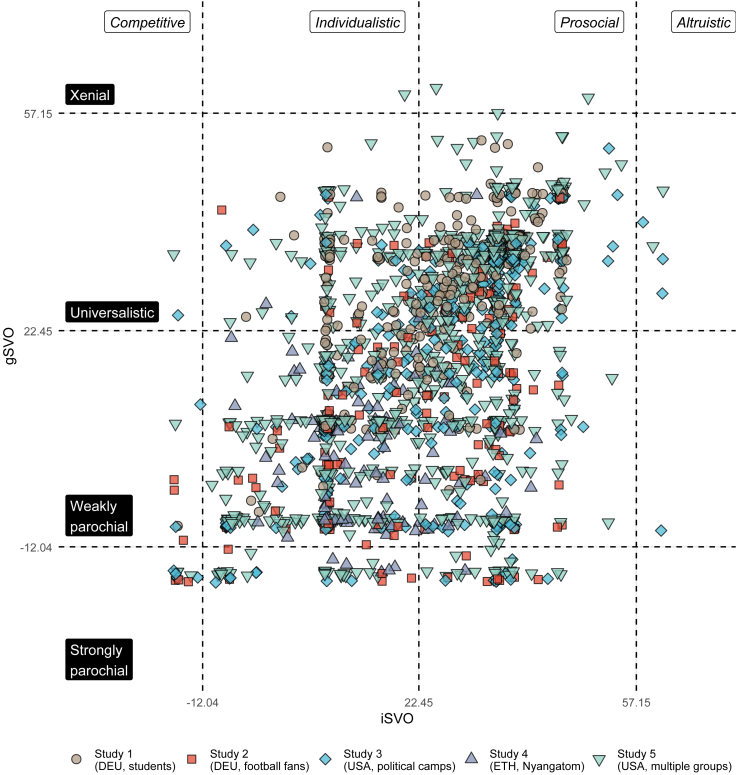
All iSVO and gSVO data points collected across the five studies (N=2,156) Multiple observations per participant possible; mild jitter was added for better display (up to ± 0.5 units in each dimension). Noteworthy observations are: (*i*) almost the entire space of possible iSVO/gSVO combinations is populated; (*ii*) the bulk of all iSVO/gSVO combinations falls into the four categories of “universalistic/individualistic” (8%), “weakly parochial/individualistic” (29%), “universalistic/prosocial” (43%), and “weakly parochial/prosocial” (14%).

Figure 1 maps the resulting two-dimensional preference space for iSVO and gSVO and shows all data points we collected across studies 1–5. Note that iSVO and gSVO are continuous measures; additionally, Figure 1 also shows the theoretically derived thresholds separating four discrete preference types along each dimension. For iSVO, we adopt the canonical labels for these types from Murphy et al.^36^ For gSVO, we suggest the labels “xenial,” “universalist,” “weakly parochial,” and “strongly parochial.”^12^^,^^38^

## Results

### Study 1: Psychometric validation among German students

To validate the psychometric properties of our measurement toolkit, we recruited German university students to participate in an online study with two measurement occasions, T1 and T2, two weeks apart from each other (*N* = 156 participants completed both measurements). In both measurements, we assessed participants’ individual-level social preferences using the iSVO measure as well as their group-level social preferences using the gSVO measure to evaluate both measures’ test-retest reliability. As the marker of in- and out-group membership, we used students’ faculty membership (engineering vs. business). We further elicited several established measures to test their relations with the new gSVO measure, aiming to additionally test its convergent and construct validity (see STAR Methods).

Results showed considerable and comparable stability of the levels of both iSVO and gSVO across measurement occasions: *r*(154) = 0.66 (95% CI [0.56, 0.74], *p* < 0.001) with the continuous score and 83.3% test-retest classification to the same preference type for iSVO, and *r*(154) = 0.58 (95% CI [0.46, 0.67], *p* < 0.001) and 79.5% for gSVO. Regarding convergent validity, as expected, gSVO (at T1, with larger values indicating higher xenialism) was negatively associated with stated motivations to maximize the ingroup member’s absolute and relative payoff (“max ingroup,” *r*(169) = -0.57, 95% CI [-0.67, -0.46], *p* < 0.001; “max rel ingroup,”: *r*(169) = -0.33, 95% CI [-0.46, -0.19], *p* < 0.001), and positively associated with motivations to minimize differences in payoffs between the ingroup and the outgroup member (“min diff”: *r*(169) = 0.26, 95% CI [0.11, 0.39], *p* < 0.001) and to maximize their joint payoffs (“max joint ingroup,” *r*(169) = 0.26, 95% CI [0.11, 0.39], *p* < 0.001; “max joint outgroup,” *r*(169) = 0.36, respectively).^39^ We also found a medium-sized correlation, *r*(169) = 0.30 (95% CI [0.16, 0.43], *p* < 0.001) of gSVO with Honesty-Humility from the HEXACO personality model,^40^ which captures individual differences in being fair and genuine toward others, and with social dominance orientation, *r*(169) = -0.25 (95% CI [-0.39, -0.11], *p* < 0.001), which captures individual differences in the support for social hierarchies.^41^^,^^42^ Overall, these results support our measure’s test-retest reliability, convergent validity, and construct validity (see supplemental information for further analyses and correlations with trait measures).

Moreover, with respect to assumption A1, we observed a considerable correlation of iSVO and gSVO in this sample (T1: *r*(169) = 0.54, 95% CI [0.43,0.64], *p* < 0.001, *n*_*T*1_ = 171; T2 *r*(154) = 0.43, 95% CI [0.29, 0.55], *p* < 0.001, *n*_*T*2_ = 156), but also meaningful variance along both dimensions and in the resulting distribution of preference types. This suggests that by including both iSVO and gSVO in our new measurement toolkit, we are able to assess and dissociate individual- and group-level social preferences.

### Studies 2 and 3: Predicting outgroup harm in intergroup conflict games

To test whether iSVO and gSVO both independently predict behavior in intergroup conflicts (and thus show predictive validity), i.e., assumption A2, we conducted two online experiments, sampling participants from natural groups with strong ingroup identification and between-group hostility in contemporary industrialized societies.

#### Study 2: German football fans

Participants in Study 2 were *N* = 193 supporters of one of two German first-league football clubs rivaling in a long-standing local derby. Football is one of the most popular sports in Germany and many fans are highly committed to their club. Many organized fan groups exist and hostilities among members of conflicting supporter groups are common.^43^ For Study 2, we invited fans of Borussia Dortmund and Schalke 04, two clubs which have one of the fiercest derby traditions.

Participants completed the iSVO and gSVO measures and further played a monetarily incentivized intergroup conflict game, the “intergroup prisoner’s dilemma (IPD).”^44^ People who supported the same club were assigned to three-person groups, each of which interacted with another three-person group composed of supporters of the opponent club. Participants were asked to distribute a monetary endowment between a private pool, benefiting only themselves, and a between-group pool, benefiting ingroup members while simultaneously harming outgroup members. Contributions to the between-group pool thus model engagement in intergroup conflict. Note, however, that conflict engagement in the IPD does not fully reveal an individual’s preferences: contributions to the between-group pool can be the result of high iSVO, low gSVO, or any suitable combination of the two—see supplemental information for a formal breakdown of this fact.

**Figure 2:**
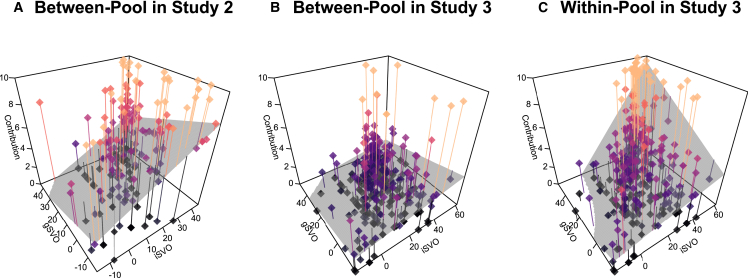
Costly individual contributions to group-beneficial investment options (z axis) as a function of iSVO (*x* axis) and gSVO (*y* axis) Dots represent observations, lines indicate vertical distance to respective regression prediction plane. (A) Contributions to the between-pool in Study 2; (B and C) Contributions to the between- and within-pool, respectively, in Study 3. The gray planes indicate predictions by the respectively best fitting regression model from Table S5. Importantly, iSVO and gSVO interact significantly and positively in explaining contributions to the “peaceful” within-group pool in Study 3, but not in explaining contributions to the outgroup harming between-pool.

Figure 2 (panel A) plots participants’ contributions to the between-group pool as a function of iSVO and gSVO. When we regress contributions to the between-group pool on iSVO, gSVO, and relevant controls (age and gender), both iSVO (unstandardized *B* = 0.08, SE = 0.02, *p* < 0.001) and gSVO (unstandardized *B* = −0.06, SE = 0.02, *p* < 0.001) show independent effects (*R*^2^ = 0.12, *F*(4,188) = 6.51, *p* < 0.001) and no significant interaction. Accordingly, iSVO and gSVO predict choices that simultaneously harm outgroup members while benefiting ingroup members at a cost to the individual better jointly than each of the preferences does alone (model comparison *F*’s >*rbin*16.1, *P*’s < 0.001). Moreover, in line with theory, participants with relatively higher levels of iSVO and lower levels of gSVO were particularly inclined to engage in intergroup conflict (for full regression models, see supplemental information, Table S5).

Thus, corroborating assumption A2, Study 2 supports that iSVO and gSVO both contribute to explaining conflict engagement. With respect to assumption A1, we again observe a correlation of iSVO and gSVO (*r*(191) = 0.46, 95% CI [0.34,0.56], *p* < 0.001), but also more variance along both dimensions and in the resulting distribution of preference types.

#### Study 3: US political camps

Importantly, in the game used in Study 2, participants can only benefit their ingroup when simultaneously harming the outgroup; thus, the game requires participants to choose between selfish and strongly parochially altruistic *behavior*. Theoretically, though, and in more realistic settings, group members may also be able to benefit the ingroup without having to harm the outgroup, that is, to engage in *weakly* parochially altruistic behavior.^12^ Study 3 therefore sought to replicate and extend the findings from Study 2 to contexts where participants can also benefit their ingroup peacefully.

The between-group conflict game in Study 3, the “IPD-maximizing difference (IPD-MD),” accordingly adds a third option allowing participants to choose between behaviors representing weakly or strongly parochially altruistic behavior. Herein, the peaceful, ingroup-beneficial option consists of costly contributions to a within-group pool which produces a public good for the ingroup but does not impose any costs on the outgroup^45^; also see STAR Methods. Note that, based on our two-dimensional preference framework, and relative to Study 2, we now can expect a behavioral separation of preference types: particularly those individuals who score high on both iSVO *and* gSVO should contribute to the within-group pool. In contrast, individuals who score high on iSVO but low on gSVO should aim to harm the outgroup by contributing to the between-group pool (also see supplemental information).

Participants were *N* = 425 US Americans identifying as supporters of either the Democratic or the Republican party. These parties are characterized by sharp ideological divides along the liberal-conservative spectrum, which addresses issues such as foreign policy, climate protection, and healthcare. These groups thus constitute strong group identities with between-group hostility.^46^^,^^47^ All participants were assigned to three-person groups composed of supporters of the same party that interacted with another group composed of supporters of the opposing party.

Replicating our main result from Study 2, we again found that both iSVO (unstandardized *B* = 0.02, SE = 0.01, *p* = 0.008) and gSVO (unstandardized *B* = −0.03, SE = 0.01, *p* < 0.001) predicted higher contributions to the between-group pool (*R*^2^ = 0.04, *F*(4,420) = 4.89, *p* < 0.001; for full regression models, see supplemental information, Table S5). That is, participants scoring high on iSVO and low on gSVO again showed the highest level of conflict engagement. Moreover, both iSVO and gSVO predicted peaceful within-group cooperation consistent with theory: for both higher levels of iSVO (unstandardized *B* = 0.08, SE = 0.01, *p* < 0.001) and higher levels of gSVO (unstandardized *B* = 0.02, SE = 0.01, *p* = 0.01) contributions to the within-group pool were higher (*R*^2^ = 0.21, *F*(2,422) = 57.39, *p* < 0.001). Beyond this, iSVO and gSVO showed a significant, positive interaction in explaining this behavior (see supplemental information, Table S5); Figure 2 visualizes our results for Studies 2 and 3.

Thus, corroborating assumptions A1 and A2 of the parochial altruism model once more, Study 3 provides evidence that iSVO and gSVO both contribute to explaining conflict engagement; with respect to assumption A1, we again observe a correlation of iSVO and gSVO (*r*(423) = 0.54, 95% CI [0.47, 0.61], *p* < 0.001) as well as substantial variance along both dimensions and in the resulting distribution of preference types. Crucially, the ability of our two-dimensional measure to explain the behavioral separation of preference types in the IPD-MD demonstrates the measure’s superiority relative to previous one-dimensional approaches, which confounded individuals’ individual- and group-level social preferences.

### Studies 4 and 5: Testing for preference change in different conflict settings

Having found quite robust support for assumptions A1 and A2 as derived from parochial altruism models more generally, we next move to quasi-experimental tests of predictions P1 and P2 as put forward by Choi and Bowles^14^ in particular. To this end, we measured iSVO and gSVO in contexts of real-world intergroup conflict, testing whether differences in conflict exposure or perceived conflict intensity are systematically associated with different levels of iSVO and gSVO in the respective participants. Truly experimental manipulations of real-world conflict exposure or intensity are ethically prohibited and methodologically unfeasible. Therefore, we resort to quasi-experimental manipulations in studies 4 and 5, thus maintaining external validity with respect to participants’ involvement in real-world intergroup conflict. We consider this indispensable, as any short-term experimental manipulation would unlikely allow for sufficient time for participants’ preferences to adapt to the context.

#### Study 4: Exposure to real-world violent intergroup conflict among the Nyangatom in Ethiopia

We conducted a laboratory-in-the-field study with members of the Nyangatom, a small-scale society in Ethiopia, living near the borders with Kenya and south Sudan. The Nyangatom regularly engage in cross-border conflicts with other ethnic groups, often resulting in fatalities.^32^^,^^48^^,^^49^ Indeed, most Nyangatom have been directly affected by violent conflict, such as having a family member killed or injured. Yet, there are considerable differences in the extent of direct conflict exposure depending on where individuals live. We leveraged this heterogeneity to quasi-experimentally manipulate conflict exposure across participants.

We sampled Nyangatom participants from both a border area in close proximity to hostile groups’ settlements (*n*_*B*_ = 61) and an interior area several days of walking from neighboring groups (*n*_*I*_ = 50). Participants from the border area reported higher involvement in violent conflicts in the previous six months than participants from the interior area (54% vs. 16%, test of proportions *χ*^2^ = 15.53, *p* < 0.001). We assessed participants’ iSVO with an unknown other Nyangatom as the recipient. Participants’ gSVO was elicited with an unknown other Nyangatom and an unknown member of a specific, hostile outgroup as the recipients.

**Figure 3:**
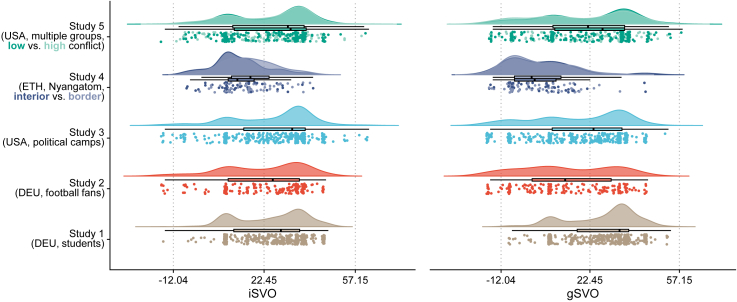
Distributions of all iSVO and gSVO data points collected across the five studies Broken down by subgroups for studies 4 and 5 (N=2,156, multiple observations per participant possible); mild jitter was added for better display (up to ± 0.1 units); boxplots follow Tukey's definitions and show the median, the first and the third quartiles (box), and 1.5 of the interquartile range (whiskers). Significant differences were found for: (*i*) iSVO levels in Study 4 and (*ii*) gSVO levels in Study 5; see main text for further details.

Contrary to prediction P2, we found no difference in gSVO when comparing individuals from border and interior areas (independent samples two-sided *t* test: *t*(109) = 0.36, *p* = 0.717, *d* = 0.07). In fact, the vast majority of our participants in this study, 100 vs. 11, fell into the same gSVO class of “weakly parochial” (also see Figure 3). Individuals from the border area did show higher levels of iSVO, though (independent samples two-sided *t* test: *t*(109) = 2.11, *p* = 0.038, *d* = 0.40). Furthermore, a significant two one-sided test (TOST) equivalence test (*t*(98.41) = −1.67, *p* = 0.049), for *d* = 0.4 suggests that if gSVO differed for border and interior participants, this difference did not reach the magnitude of the observed difference in iSVO.

Nonetheless, when we exploratively regressed our individual-level manipulation check variable of self-reported conflict involvement on iSVO, gSVO, and their interaction, controlling for approximate age, we found that iSVO (*B* = 0.070, SE = 0.024, *p* = 0.004), gSVO (*B* = 0.076, SE = 0.034, *p* = 0.024), and their interaction (*B* = −0.004, SE = 0.002, *p* = 0.018) were associated with conflict involvement (see supplemental information for details). In particular, our regression results imply that participants with high levels of gSVO, i.e., the relatively more universalistic individuals, show a reduced association of iSVO with conflict involvement compared to relatively more parochial individuals. In other words, the association between iSVO and conflict involvement was weaker among relatively more universalistic individuals.

Thus, supporting prediction P1 in this sample, we replicated earlier findings of a positive link between conflict exposure and ingroup prosociality.^29^^,^^30^^,^^31^ Moreover, unlike in studies 1–3, iSVO and gSVO were not correlated in our Nyangatom participants (*r*(109) = -0.01, *p* = 0.938). This provides additional evidence for the independence of the two preference dimensions, thus again supporting assumption A1.

With respect to P2, our quasi-experimental manipulation did not uncover systematic differences in gSVO between more (vs. less) conflict-exposed participants. Nonetheless, the interaction we uncovered indicates that gSVO could moderate the known link between ingroup prosociality and conflict exposure. Alternatively, given the correlational nature of our evidence on individual conflict involvement in Study 4, this pattern could be explained by self-selection into conflict by relatively more parochial and more prosocial individuals, i.e., parochial altruists, which would corroborate A2.

At the same time, almost all of our participants in Study 4 showed high levels of parochialism toward members of the specific outgroup we had selected. It is important to note that our border vs. interior comparison aimed to quasi-experimentally manipulate actual conflict involvement (participation), whereas gSVO may be more sensitive to perceived conflict intensity. Thus, although participants from the interior area reported lower involvement in violent conflicts in the previous six months than participants from the border area, they might have perceived the conflict with the target outgroup just as intensely, irrespective of where they lived at the time. This distinction between conflict exposure and perceived conflict intensity potentially explains why gSVO did not vary systematically with conflict exposure in Study 4.

#### Study 5: Perceived intensity of conflict between groups in the US

To overcome this limitation of Study 4, we followed up with our preregistered Study 5, in which we quasi-experimentally manipulated the perceived intensity of conflict between natural groups. This allows us to test if iSVO and gSVO systematically vary with perceived conflict intensity, independent of an individual’s degree of exposure to a conflict.

In Study 5 we presented *N* = 236 US-Americans with five pairings of groups (men/women, meat-eaters/non-meat-eaters, Republicans/Democrats, Christians/Muslims, and supporters of pro-life/pro-choice). Participants who did not identify with exactly one of the groups in each pair as their respective ingroup were excluded from further participation. Participants then rated the degree of conflict they perceived between the groups in each pair and their degrees of identification with the respective ingroups.

Our quasi-experimental manipulation of conflict intensity consisted in selecting two pairs of groups for each participant based on their individual ratings: the pair with the highest and the pair with the lowest perceived degree of intergroup conflict. For each of these two pairs per participant, we elicited iSVO and gSVO. That is, each participant made decisions in two group contexts that were selected based on the respective participant’s own perceptions of conflict intensity: one high-conflict context and one low-conflict context.

Our manipulation of perceived between-group conflict intensity resulted in a significant difference in gSVO scores in the hypothesized direction (*M*_*high*_ = 18.54 vs. *M*_*low*_ = 21.18, two-sided paired *t* test: *t*(235) = 2.18, *p* = 0.03, *d* = 0.14; Figure 3). We did not find any significant differences in iSVO in Study 5, though (*M*_*high*_ = 24.72 vs. *M*_*low*_ = 25.14, two-sided paired *t* test: *t*(235) = -0.61, *p* = 0.54, *d* = 0.06; also see supplemental information). Furthermore, a significant TOST equivalence test (*t*(235) = -1.66, *p* = 0.05), for *d* = 0.15 suggests that if iSVO differed for participants’ “high conflict” vs. “low conflict” pairings, this difference unlikely reached the magnitude of the observed difference in gSVO. As in studies 1–3, iSVO and gSVO showed substantial correlations at both high and low levels of perceived conflict (high: *r*(234) = 0.50, 95% CI [0.40, 0.59], *p* < 0.001; low: *r*(234) = 0.62, 95% CI [0.53, 0.69], *p* < 0.001).

## Discussion

Across five studies assessing original data in different cultural contexts and from natural groups with ongoing conflicts of varying intensities, we find that our two-dimensional measurement toolkit of iSVO and gSVO characterizes individuals’ social preference types better than previous one-dimensional metrics. Specifically, our findings corroborate two central assumptions of parochial altruism models: individuals’ preferences are indeed separable along the two dimensions (A1) and both dimensions meaningfully and independently contribute to explaining intergroup behavior (A2). Table 1 succinctly summarizes our studies’ features and main findings.

**Table 1:** Overview of the key features of all studies reported in this paper and brief summaries of main findings

Study	Sample	Grouping	Method	Findings
1	students, DEU	field of study	correlational, two waves	iSVO and gSVO have sound psychometric properties
2	football fans, DEU	fandom for Borrussia Dortmund vs. Schalke 04	correlational, IPD game	iSVO and gSVO independently predict contributions in the IPD game
3	Prolific, USA	preference for Democrats vs. Republicans	correlational, IPD-MD game	iSVO and gSVO independently predict contributions and explain behavioral separation of types in IPD-MD relative to IPD game
4	Nyangatom, ETH	border vs. interior area	quasi-experimental, between-participants	iSVO higher in border region, no zero-order difference in gSVO, but gSVO might moderate association of iSVO and conflict involvement
5	Prolific, USA	several groups, high vs. low perceived conflict	quasi-experimental, within-participants	gSVO lower for high-conflict pairings, no zero-order difference in iSVO

With regard to the dynamics of preference change theoretically predicted by Choi and Bowles,^14^ our results provide a more nuanced picture. If the original model was correct, individuals exposed to conflict should show higher altruism (P1) and higher parochialism (P2), and vice versa for exposure to peace. Supporting P1 but not P2 within a field setting, we find in Study 4 that gSVO does not vary as sensitively with exposure to violent intergroup conflict as iSVO does. However, our successful attempt to manipulate gSVO quasi-experimentally in Study 5 still supports the idea that (perceived) conflict intensity does affect gSVO. That said, when interpreting the apparent differences between the two studies, it is important to keep in mind that they measured different facets of conflict experience—actual conflict participation in Study 4 versus perceived conflict intensity in Study 5. Future research could investigate how these dimensions interact and jointly shape parochial and prosocial preferences.

Interestingly, in Study 5 we also observe that gSVO varies within-person for different in- and out-group pairings, while iSVO does not. This finding is incommensurable with the original parochial altruism model, which does not allow for varying attitudes toward different in- and out-groups, i.e., in different conflicts. Moreover, our finding that iSVO was generally higher in our samples with lower exposure to intergroup violence and unaffected by our manipulation of conflict intensity suggests that P1 does not hold in general, but needs to be interpreted in narrower terms. Our toolkit allows to better connect parochial altruism models with empirical data, providing new opportunities for theory development and evidence-based refinement.

### Limitations of the study

Our research comes with some limitations that warrant further investigation. First, as the individual levels of both iSVO and gSVO are endogenous, drawing causal inferences is naturally difficult. Studies 4 and 5 try to overcome this limitation partly by employing quasi-experimental manipulations. Future research may further investigate changes in both individual-level altruistic preferences and group-level parochial preferences as a function of exogenous shocks to the conflict environment.

Second, the conflict games utilized in studies 2 and 3, while well-established, represent highly stylized conflict scenarios. For example, these paradigms do not provide participants with the option of engaging in peaceful intergroup cooperation—only selfish behavior or cooperation within one’s own group—as alternatives to harmful conflict engagement. However, low levels of parochialism (gSVO) and high levels of altruism (iSVO) should also predict constructive intergroup interactions, including cooperation. Future research should therefore explore the predictive value of iSVO and gSVO in relation to positive intergroup behavior using game paradigms that are specifically designed to capture such outcomes.^50^

Third, although we included several established individual difference measures previously linked to conflict behavior in Study 1—e.g., social dominance orientation^41^^,^^42^—to assess the convergent and construct validity of the gSVO measure, we did not include these variables as predictors of conflict behavior in studies 2 and 3. Future research should therefore examine whether gSVO continues to predict conflict behavior when controlling for such alternative explanatory variables.

Fourth, while our study examines the prevalence and impact of social preferences across diverse samples and group contexts, future research should further assess the generalizability of these findings across a broader range of geographical and psychological settings and should investigate the proximate functioning of the mechanisms underlying preference change more closely. For example, it would be valuable to measure both individual- and group-level social preferences across different cultural contexts—e.g., with different levels of past and current conflict exposure—and with respect to multiple group identities within the same participants. Such research could help disentangle within-versus between-participant determinants of these preferences and their associated outcomes.

Fifth and relatedly, the literature suggests that conflict behaviors are influenced not only by individual preferences but also by beliefs about others’ behaviors^25^ and situational factors.^51^^,^^52^^,^^53^^,^^54^ While we chose to exclusively focus on individual- and group-level social preferences in the present studies, a possible next step for future research is to explore how these preferences relate to different beliefs and how their effects may depend on relevant situational features, including information obtained about in- and out-group members through repeated interactions. Moreover, our studies do not directly address how social identity processes influence—or are influenced by—individual differences in iSVO and gSVO (but see supplemental information for initial insights). Exploring these relations will be essential to advance theoretical development and integration.

### Conclusion

In conclusion, the studies presented in this paper add two main insights to the literature on parochial altruism. For one, individual-level altruistic preferences (i.e., iSVO) and group-level parochial preferences (i.e., gSVO) need to be elicited and modeled separately in future micro-level work on intergroup conflict, as they differentially contribute to explaining individual behavior in this context. This is crucial, as prior research relied exclusively on measures of individual-level social preferences and conflict behavior, inferring group-level social preferences only indirectly.

Second, iSVO and gSVO differ in their dynamics and their scopes: our results suggest that iSVO changes with conflict exposure but not, or much less, with conflict intensity, while gSVO changes with (perceived) conflict intensity but not, or much less, with conflict exposure. The details of this intricate interplay of individual- and group-level preferences with conflict exposure and perceived conflict intensity need to be more systematically scrutinized in future work. The present paper provides the psychometric tools required for this task to enrich and stimulate future research on the very nature of human intergroup conflict.

## Resource availability

### Lead contact

Requests for further information and resources should be directed to and will be fulfilled by the lead contact, Hannes Rusch (h.rusch@csl.mpg.de).

### Materials availability

This study did not generate new unique reagents.

### Data and code availability

• The anonymized datasets for all studies have been deposited at OSF and are publicly available via DOI: https://doi.org/10.17605/OSF.IO/RG6VY.
• The associated code needed to reproduce the results reported in this article has been deposited at OSF and is publicly available via DOI: https://doi.org/10.17605/OSF.IO/RG6VY.
• The study materials (i.e., participant instructions in English) have been deposited at OSF and are publicly available via DOI: https://doi.org/10.17605/OSF.IO/RG6VY.
• Any additional information required to reanalyze the data reported in this article is available from the lead contact upon request.

## Acknowledgments

We thank Daniel Balliet, Stefan Pfattheicher, Manvir Singh, several anonymous reviewers, and the audiences at ICSD 2017 Taormina, the ANR GROUP workshop “In-group favoritism in intergroup conflicts,” and the Max Planck Summer School on the Political Economy of Conflict and Redistribution for helpful comments. All remaining mistakes are ours. This research received financial support from the “Excellence Initiative” (ZUK II) of the 10.13039/501100001659German Research Foundation (DFG) at RWTH Aachen University (to R.B.) and from The Eric M. Mindich Research Fund for the Foundations of Human Behavior as well as the 10.13039/100007229Mind Brain and Behavior Interfaculty Initiative at Harvard University (to L.G.).

## Author contributions

Conceptualization, R.B., H.R., and I.T.; data collection, R.B., L.G., and I.T.; formal analysis, H.R.; statistical analysis, H.R. and I.T.; funding acquisition: R.B. and L.G.; methodology, all authors; visualization, H.R.; writing – original draft, R.B. and H.R.; writing – review & editing, all authors. The authors consider their contributions equal and are listed in alphabetical order.

## Declaration of interests

The authors declare no conflict of interest.

## STAR★Methods

### Key resources table

**Table:** 

REAGENT or RESOURCE	SOURCE	IDENTIFIER
**Deposited data**		

Study data	This paper	OSF: https://doi.org/10.17605/OSF.IO/RG6VY

**Software and algorithms**		

Analysis script	This paper	OSF: https://doi.org/10.17605/OSF.IO/RG6VY

**Other**		

Study materials	This paper	OSF: https://doi.org/10.17605/OSF.IO/RG6VY

### Experimental model and study participant details

For Studies 1–3, no ethics approval was required by the authors’ institutions, as treatment of participants was in agreement with the ethical guidelines of the German Research Foundation (*Deutsche Forschungsgemeinschaft*) and the German Psychological Society (*Deutsche Gesellschaft für Psychologie*).

Ethics approval was obtained for Study 4 (Committee on the Use of Human Subjects in Research at Harvard University, IRB protocol number: F-17615-105) and Study 5 (Departmental Review Board of the Department of Occupational, Economic, and Social Psychology at the University of Vienna, IRB protocol number: 2023/W/014A). Additional approval for Study 4 was obtained locally by the South Omo Research Center and the study complied with local laws and regulations regarding the conduct of research. The data for Study 4 were collected by LG who worked with local field assistants. This particular project did not involve local researchers. However, we have taken locally relevant citations into account in drafting our manuscript and we continuously support the Omo Valley Research Project (https://www.omovalleyresearchproject.org/) which promotes local capacity building via training of young Ethiopian scholars through scholarships and mentorship, develops collaborations with local researchers and institutions, and offers applied benefits to organizations and communities.

Across all studies, all participants gave their informed consent to participate voluntarily, were not deceived, were compensated in line with local standards, and were assured that all statistical analyses and reports would be anonymous. We confirm that, for all studies, we report all measures, conditions, and data exclusions.

#### Study 1

The study was conducted online and consisted of two measurement occasions, T1 and T2, carried out two weeks apart. The final sample consisted of *N* = 171 participants at T1 and *N* = 156 participants at T2 (final attrition rate ≈9%). They were students of a large German university (of those who completed T1: 41.5% female; age: *M* = 23.37, SD = 2.71).

#### Study 2

The study was conducted online. The final sample consisted of *N* = 193 German participants; age: = 26.37 (SD = 6.48), 22.8% (*n* = 44) female, 70.5% (*n* = 136) university students.

#### Study 3

The study was conducted online via Prolific (https://www.prolific.com/). The final sample size was *N* = 425; age: *M* = 30.71 (SD = 10.35), 42.6% (*n* = 181) female, 72.7% (*n* = 309) with college/university or higher degree.

#### Study 4

This study was conducted through individual in-person sessions, each involving one participant and the experimenter. Participants were *N* = 111 male Nyangatom. To ensure considerable variability in individuals’ conflict exposure, we sampled from two inhabited areas of Nyangatom. For the high-conflict area, we selected *n* = 61 participants living in the Kibish region near Lokorhlam. For the low-conflict sample, we randomly selected *n* = 50 participants living in the Omo River area near the town of Kangaten. The approximate age of the participants (estimated by the experimenter) was *M* = 26.17 (SD = 7.28, range = [18,50]).

#### Study 5

The study was conducted online via Prolific (https://www.prolific.com/). The final sample size was *N* = 472 participants, all from the U.S.; age: = 45.05 (SD = 14.42), 50.0% (*n* = 236) female, 71.8% (*n* = 339) had college/university degree. Study 5 was preregistered via aspredicted.org (ID: 140998). This study had two conditions, ‘perceived conflict’ and ‘identification’, each with *N* = 236 participants. We only describe the results of the ‘perceived conflict’ condition in the main text. Procedures and measures for both conditions are described below. Results of the ‘identification’ condition are reported in supplemental information.

### Method details

#### Study 1

##### Sample size and recruitment

A required sample size of *N* = 175 was determined via *a-priori* power analysis,^55^ aiming to detect correlations down to *r* = 0.21 (based on^56^) between preference and validation measures (*α* = 0.05, 1-*β* = 0.80). To compensate for potential exclusions, 196 participants started the study at T1; 179 completed T1 and 164 completed both T1 and T2 (attrition rate ≈16%). We further excluded 8 participants who (i) failed to correctly respond to one or more comprehension questions, (ii) completed the HEXACO inventory in less than 2 min, or (iii) indicated having not fully understood all tasks, assessed via self-report item (“I have understood all tasks.”). Results are robust to including the excluded participants (as can be checked in our replication materials available via DOI: https://doi.org/10.17605/OSF.IO/RG6VY). Data were collected during June and July 2016.

##### Compensation

All participants received a flat fee of EUR 8 for completion of both parts of the 40-min study. Additionally, one-third of participants (*n* = 55) was randomly selected for an additional bonus payment based on the decisions made during the study (see below); bonuses ranged from EUR 8.70 to 11.50 (*M* = 10.20, SD = 0.74).

##### Group identification

Participants were students from either the engineering or business faculty. To ensure that individuals identified with their ingroup (i.e., respective faculty), at T1 we used four items measuring participants’ strength of group identification devised by Doosje et al.^57^ We computed the responses’ mean value (Cronbach’s *α* = 0.83).

##### iSVO and gSVO: Standard measures

At both T1 and T2, participants completed the iSVO and gSVO slider measures; see Methods S1 (‘iSVO/gSVO slider measures’) for measure details. Measurement order was counter-balanced across participants. Tokens allocated via iSVO were transformed into money at conversion rate 100 tokens = EUR 0.50. All participants completed iSVO as allocator. We later randomly determined whether a participant was paid as allocator or recipient. For this, participants were randomly matched into pairs. Exactly one of the six iSVO items was paid out. Similarly, all participants completed gSVO as (third-party) allocator. We later randomly determined whether they were paid as allocator, ingroup recipient, or outgroup recipient. When participants were selected as allocator, they received a fixed payoff of EUR 2.00. When selected as recipient, their payoff was determined by the choices of another randomly matched ingroup or outgroup member. Exactly one of the six gSVO items became payoff-relevant.

##### iSVO and gSVO: *Coin measures*

At T2, we additionally used adapted coin-versions of iSVO and gSVO (shorthand: ‘iSVOcoin’ and ‘gSVOcoin’). We developed these for populations with low numeracy (as in Study 4). See supplemental information for details.

##### Between-group orientations (‘BGO’)

At T1, we assessed the BGO measure,^39^ which comprises 10 items asking participants to allocate monetary tokens between an ingroup member and an outgroup member (similar to gSVO). Each of the seven choice options represents a distinct motivation: maximization of the ingroup member’s absolute payoff (‘max ingroup’), maximization of the ingroup member’s payoff relative to the outgroup member (‘max rel ingroup’), minimization of the payoff difference between ingroup and outgroup member (‘min diff’), maximization of the outgroup member’s absolute payoff (‘max outgroup’), maximization of the outgroup member’s payoff relative to the ingroup member (‘max rel outgroup’), maximization of the ingroup and outgroup members’ joint payoff favoring the ingroup member (‘max joint ingroup’), and maximization of the ingroup and outgroup members’ joint payoff favoring the outgroup member (‘max joint outgroup’). The total number of choices in line with a specific motivation (ranging from 0 to 10) indicates the importance of that motivation.

In the experiment, the conversion rate for tokens to money was 100 tokens = EUR 0.50. All participants completed BGO as allocator. It was later randomly determined whether they were paid as allocator, ingroup recipient, or outgroup recipient. Allocators received a fixed payoff of EUR 1.00. Recipients received the payoff resulting from the allocator’s decision. Exactly one item became payoff-relevant.

##### HEXACO

At T1, participants completed the German 60-item HEXACO Personality Inventory-Revised (Ashton and Lee^58^; https://www.hexaco.org. HEXACO-60 comprises 10 items assessing six personality dimensions: honesty-humility, emotionality, extraversion, agreeableness, conscientiousness, and openness to experience. Items are answered on a 5-point Likert-type scale ranging from 1 = “strongly disagree” to 5 = “strongly agree”. The 10 items for each personality trait were averaged (after recoding reversed-scored items) to form independent subscales (Cronbach’s *α*: honesty-humility = 0.79, emotionality = 0.75, extraversion = 0.83, agreeableness = 0.70, conscientiousness = 0.76, openness to experience = 0.73).

##### Social Dominance Orientation (‘SDO’)

At T1, we used a German translation^41^ of the SDO scale^42^ to assess participants’ SDO. The 12 scale items assess individuals’ preference for group-based hierarchies within a social system on a 5-point Likert-type scale ranging from 1 = “do not agree at all” to 5 = “do fully agree”. Cronbach’s *α* for the SDO scale (i.e., mean value of participants’ responses on the 12 items) was 0.89.

##### Group authoritarianism (‘GA’)

Participants’ beliefs about the appropriate relationship between groups and their individual members was assessed at T1 based on the GA measure.^59^ The 12 items of this scale are answered on a 6-point Likert-type scale from 1 = “do not agree at all” to 6 = “do fully agree”. Cronbach’s *α* for the scale (mean value across the 12 responses) was 0.84.

##### Identification with all Humanity (‘IWAH’)

We measured participants’ IWAH at T1, conceptualized as an individual’s concern for global harmony and equality. We used the German translation^60^ of the original 9-item measure.^61^ Items are answered on a 5-point Likert-type scale with varying labels, e.g., “How close do you feel to each of the following groups?” (groups: “people in my community”, “Germans”, “people all over the world;” scale ranging from 1 = “not at all close” to 5 = “very close”). For each of the three levels (i.e., personal, own country, people all over the world), we computed the mean value. Cronbach’s *α* was 0.79 for the personal level as well as for the country level, and 0.83 for the global level.

##### Procedure

Upon invitation, participants were informed that the study consisted of two parts, with a time lag of two weeks in between. We assessed pseudonymized codes that participants had to provide at both measurement occasions to allow matching their data. After completion of T2, participants could obtain their individual payment using their pseudonymized codes.

To elicit group-level preferences, we used natural groups. We excursively recruited social and business sciences students and engineering sciences students. At T1, participants were asked to which of the two groups they belonged. We then assessed their identification with their respective ingroup. We referred to this group membership (and the corresponding ingroup and outgroup categories) in the gSVO, gSVOcoin, and BGO measures.

Participants first completed iSVO and gSVO in counter-balanced order (both at T1 and T2). Afterward, they completed all other measures in randomized order (i.e., HEXACO-60, SDO, GA, and IWAH at T1; iSVOcoin and gSVOcoin at T2). After completion of all measures at T1, participants answered several questions on how seriously they participated in the study. Feedback about payoffs (and thus information about other participants’ decisions) was only given upon completion of T2.

#### Study 2

##### Sample size and recruitment

Sample size was determined based on *a-priori* power analysis,^55^ assuming a small to medium-sized correlation (*r* = 0.21; based on^56^) between the social preference measures and contribution behavior in the between-group conflict game (*α* = 0.05, 1-*β* = 0.80). This resulted in a required sample size of 175 participants. We thus recruited 194 fans of the German soccer clubs Borussia Dortmund and Schalke 04 via supporter clubs, social network groups, and university mailing lists. One participant had to be excluded due to indicating insufficient German language skills. Results are robust to including the excluded participant (as can be checked in our replication materials). Data were collected during September 2016.

##### Compensation

Thirty participants were randomly selected to receive behavior-contingent payment. Lottery winners were informed via email, payments were realized via bank transfer. Payoffs ranged from EUR 7.50 to EUR 28.80 (*M* = 17.74, SD = 5.32).

##### iSVO and gSVO

Participants completed the iSVO, with the recipient being an “unknown other person.” For gSVO, recipients were a “supporter of [ingroup team]” and a “supporter of [outgroup team]”. Tokens allocated were transformed into money at conversion rate 100 tokens = EUR 10.00. It was randomly determined whether participants were paid as allocator or recipient in iSVO, and as allocator, ingroup recipient, or outgroup recipient in gSVO. Allocators in gSVO received a fixed payoff of EUR 10.00. One item of each iSVO and gSVO was randomly selected for payment.

##### Between-group conflict game

To measure participants’ engagement in between-group conflict, they played the Intergroup Prisoner’s Dilemma (‘IPD’) game.^44^ Participants were assigned to three-person groups matched with other three-person groups. They received an endowment of 10 monetary tokens (worth EUR 10.00) and could decide how many tokens (if any) to contribute to a private pool, benefiting only themselves, and to a between-group pool, benefiting all ingroup members but harming all outgroup members (see supplemental information for a formal description).

##### Further measures

We used the same group identification measure as in Study 1.^57^ Cronbach’s *α* was 0.82. Results are reported in supplemental information.

##### Procedure

Participants were invited to participate in an online study on decision making involving soccer fans. Only supporters of Borussia Dortmund or Schalke 04 were eligible for participation. After the group identification measure, participants completed iSVO and gSVO in counterbalanced order, followed by the IPD game. Before participants made their contribution decision in the IPD, they received a numerical example of potential game outcomes and had to correctly answer three comprehension questions about the rules of the game. At the end, participants were free to leave their email address to participate in the lottery for payment.

#### Study 3

##### Sample size and recruitment

Based on previous research,^26^^,^^47^^,^^62^ we expected only small contributions to the between-group conflict pool in the IPD-MD. Therefore, we aimed to double sample size relative to Study 2. We recruited *N* = 447 participants but had to exclude *n* = 22 participants who indicated having not fully understood all tasks, as assessed through a self-report item (“I have understood all tasks.”). Results are robust when including these participants (as can be checked in our replication materials). Data were collected during February 2016.

##### Compensation

All participants received a fixed payment of USD 1.30 for completing the 15-min study. Additionally, 140 participants were randomly selected to receive behavior-contingent payment based on iSVO, gSVO, and the IPD-MD. Payments were executed via Prolific, ranging from USD 0.89 to USD 3.26 (*M* = 2.23, SD = 0.38).

##### iSVO and gSVO

Participants completed iSVO, with the recipient being an “unknown other person” or a member of the ingroup, that is, a supporter of the same political party as the participant. This manipulation was implemented to investigate whether iSVO differs for neutral vs. ingroup recipients. It did not (see supplemental information). For gSVO, recipients were a “supporter of [political ingroup]” and a “supporter of [political outgroup]”. Tokens allocated in iSVO and gSVO were transformed into money at conversion rate 100 tokens = USD 1.00. Allocators in gSVO received a fixed payoff of USD 1.00. All other procedures regarding measurement and payment were identical to Study 2.

##### Between-group conflict game

To measure participants’ engagement in between-group conflict, they played the IPD-MD game.^45^ The game is identical to the IPD, with the exception that it adds one additional pool: keeping tokens or contributing them to the between-group pool, participants can also contribute to a within-group pool, benefiting all ingroup members without harming the outgroup (see supplemental information for a formal description).

##### Further measures

We used the same four-item measure of identification with one’s ingroup as in Study 2 to assess participants’ identification with the political group they supported. Cronbach’s *α* for the scale (mean value across the four responses) was 0.92. Results are reported in supplemental information.

##### Procedure

The procedure was the same as in Study 2, with the exception that group identification was assessed after the between-group conflict game. Furthermore, at the end of the survey, participants answered several questions on how seriously they participated in the study.

#### Study 4

##### Sample size and recruitment

We aimed to recruit as many male Nyangatom as possible within the study period. The Nyangatom area is approximately 2,600 km^2^ within Ethiopia and borders Kenya and South Sudan. The border with Kenya is marked by frequent between-group conflict primarily in the form of livestock raids that are often violent.^63^ Other areas, particularly by the Omo river, show between-group violence only very rarely due to the distance from other groups and the difficulties of traveling by foot.

We sampled participants from the Kibish region near Lokorhlam (high-conflict area) and from the Omo River area near the town of Kangaten (low-conflict area). The Kibish region is approximately two kilometers from the Kibish river and border with Kenya, and is subject to frequent livestock raids. For example, Yntiso reports nine raids that resulted in 15 deaths during an 18-month period prior to the study.^63^ In contrast, the Omo River area is only very rarely subject to between-group violence, having no raids or deaths due to between-group violence in the same 18-month period prior to the study.^63^ Data were collected during March and April 2016.

##### Compensation

Participants received a show-up fee of ETB 15.00 (about USD 0.50 at the time) and could earn additional payment depending on their iSVO and gSVO choices; the aggregated mean payment was *M* = 58.63 ETB (SD = 5.71, range = [40,68]; about USD 2.00).

##### iSVO and gSVO

Participants completed iSVOcoin and gSVOcoin. All participants completed iSVO as allocator and their own payoff depended on their own choice. All participants completed gSVO as (third-party) allocator and their own payoff was fixed to ETB 15.00, irrespective of the choices they made. Recipients of either measure did not take part as participants in the study (they were recruited separately) to reduce potential reciprocity concerns in choices. One item from each measure was randomly selected for payment.

##### Conflict exposure

We assessed participants’ personal conflict exposure by asking them about the number of conflicts (battles or raids) they had participated in the previous six months or previous three years. As the Nyangatom do not use a calendar, memory over a longer period may probably be distorted, arguably yielding the measures of conflict participation in the previous three years less reliable. Therefore, we built two groups based on participants’ responses for the previous six months: those reporting to have experienced one or more conflicts in the past six months (conflict exposure = 1) and those reporting not having experienced any conflict (conflict exposure = 0).

##### Procedure

The study was conducted in Nyangatom language. The experimenter first explained the purpose of the study and asked for participants’ consent. Each participant received verbal instructions for the tasks. It was explained that their choices may affect their own and/or other individuals’ payment (depending on the measure). The order of iSVO and gSVO was counter-balanced across participants.

For each item, participants received seven cards—each representing one option—in a fixed, horizontal layout (the order of cards was the same as in the original measure by Murphy et al.,^36^ see Table S1; we piloted the elicitation procedure also in a vertical layout with *n* = 21 participants, but this resulted in biased choices as participants tended to choose the card closest to them. Results are robust to including the pilot participants (as can be checked in our replication materials). Items were distinguishable for the experimenter by different colors (Figure S1 shows an example choice card). Participants had time to thoroughly examine the cards and then indicated their choice by pointing to one of the cards or by picking it up. The experimenter then placed the selected card in an envelope. Eventually, there were six choice cards in the envelope (one for each item of the measure). After participants completed iSVO and gSVO, they were asked about their individual conflict history.

#### Study 5

##### Sample size and recruitment

An *a-priori* power analysis for testing our hypotheses using mixed-model ANOVA (two groups, two measurements, assumed correlation between measurements: 0.5), aiming to detect a small effect of *f* = 0.1 at *α* = 0.05 and power 1-*β* = 0.99, suggested *N* = 462 participants; we aimed to recruit 500. We excluded *n* = 28 participants based on preregistered exclusion criteria. Data were collected during August and September 2023.

##### Compensation

All participants received a fixed payment of USD 1.50 for completing the 10-min study. Additionally, 100 participants were randomly selected to receive a behavior-contingent bonus payment based on iSVO and gSVO. Payments were executed via Prolific, ranging from USD 1.89 to USD 4.20 (*M* = 3.30, SD = 0.61).

##### Procedure

After providing demographic information, participants first saw the five group pairings (gender: male vs. female, political affiliation: democrat vs. republican, diet: meat vs. no-meat, views on abortion: pro-life vs. pro-choice, and religious affiliation: Christian vs. Muslim). For each pairing, participants indicated one group as the one that they identify with; participants who did not identify with exactly one of the groups for each pair were excluded from further participation. Next, participants were asked to rank the five groups they indicated according to either (*i*) the levels of conflict they perceived between the two groups within each pair (on a 5-point scale from ‘very high’ to ‘very low’) in the ‘perceived conflict’ condition, or (*ii*) these groups’ respective importance for participants’ self-conception (from ‘least central to who you are’ to ‘most central to who you are’ on a 5-point scale) in the ‘identification’ condition.

##### iSVO and gSVO

In the ’perceived conflict’ condition, participants completed two sets of iSVO and gSVO slider measures. One for the group pairing which the individual participants ranked highest on perceived conflict and one for the pairing they ranked lowest. iSVO always was elicited with the recipient being a member of the respective ingroup. Presentation order of iSVO and gSVO and high vs. low conflict contexts was counterbalanced across participants. Procedures were analogous in the ’identification’ condition. Tokens allocated in iSVO and gSVO were transformed into money at conversion rate 100 tokens = USD 1.00. Allocators in gSVO received a fixed payoff of USD 1.00.

### Quantification and statistical analysis

All analyses were executed with R, version 4.2.2. For inferential statistics, if not mentioned otherwise, we interpret all tests with *p* < 0.05 or the 95% confidence interval not including zero as statistically significant. All data exclusions and final sample sizes are reported above. All data, experimental instructions, and an executable analysis script can be accessed via https://doi.org/10.17605/OSF.IO/RG6VY.

#### Study 1

In order to assess convergent and discriminant validity of gSVO, its test-retest reliability, and its correlation with iSVO, we used standard Pearson product-moment correlation. All tests for convergent and discriminant validity are reported in Tables S2 and S3.

#### Study 2

We used ordinary least-squares regressions to test for linear effects of iSVO, gSVO, and their interaction on contributions to the between-group pool in the IPD game. We then used exact F-tests to compare the model with both iSVO and gSVO present as predictors to the two models with each of them present alone. Further, we used standard Pearson product-moment correlation to test for a correlation of iSVO and gSVO in the sample of this study. Full regression estimation results are reported in Table S4.

#### Study 3

We used ordinary least-squares regressions to test for linear effects of iSVO, gSVO, and their interaction on contributions to the within- and between-group pools in the IPD-MD game. Further, we used standard Pearson product-moment correlation to test for a correlation of iSVO and gSVO in the sample of this study. Full regression estimation results are reported in Table S4.

#### Study 4

We used a two-sample test for equality of proportions with continuity correction to test whether the shares of participants with self-reported recent conflict involvement differed between regions. To test for differences in iSVO and gSVO between regions, we used independent samples two-sided *t*-tests. To test whether any unobserved differences in gSVO would be likely to reach the magnitude of the observed difference in iSVO, we used a Welch two-sample *t* test, a null hypothesis significance test (NHST), and an equivalence test, via two one-sided tests (TOST). Further, we used standard Pearson product-moment correlation to test for a correlation of iSVO and gSVO in the sample of this study. To test for effects of iSVO, gSVO, and their interaction on self-reported conflict involvement in the previous six months, we used logistic regressions. Full regression estimation results are reported in Table S6.

#### Study 5

To test for differences in iSVO and gSVO between experimental conditions, we used paired two-sided *t*-tests. To test whether any unobserved differences in iSVO would be likely to reach the magnitude of the observed difference in gSVO, we used a Welch two-sample *t* test, a null hypothesis significance test (NHST), and an equivalence test, via two one-sided tests (TOST). Further, we used standard Pearson product-moment correlations to test for a correlation of iSVO and gSVO in the sample of this study.

## Footnotes

Supplemental information can be found online at https://doi.org/10.1016/j.isci.2025.113978.
