# Supplementary material for: Untangling altruism and parochialism in human intergroup conflict
Source: iScience. 2025 Nov 10;28(12):113978. doi: 10.1016/j.isci.2025.113978 (PMC12689215; doi:10.1016/j.isci.2025.113978)
Supplement: Document S1. Figures S1–S3, Tables S1–S6, Methods S1–S3, and Data S1–S5 [file mmc1.pdf]

**iScience, Volume *28***

## Supplemental information

# Untangling altruism and parochialism in human intergroup conflict

**Robert Böhm, Luke Glowacki, Hannes Rusch, and Isabel Thielmann**

## S1 Supplementary information and results for Study 1

### *Methods S1: iSVO / gSVO slider measures*

Table S1 shows the iSVO and gSVO sliders that we used across studies.

**iSVO.** The iSVO slider measure assesses individual-level social preferences and the novel gSVO slider measure assesses group-level social preferences. iSVO assesses individuals' general concern for others' welfare relative to own welfare. Specifically, participants make several decisions about how to allocate monetary tokens between themselves and an unknown recipient. Each of the six (primary) items consists of nine alternatives, with varying tokens for oneself (ranging between 50 and 100) and for the recipient (ranging between 15 and 100; see Table S1 for the full overview). As explained in Murphy *et al.* [S1], the sum of tokens allocated to oneself versus the recipient allows for calculating a continuous iSVO angle, ranging from *competitiveness* (iSVO $< -12.04°$; maximizing the own relative payoff and minimizing the recipient's payoff at personal cost) to *individualism* ($-12.04° \leq$ iSVO $\leq 22.45°$; maximizing the own absolute payoff), *prosociality* ($22.45° <$ iSVO $\leq 57.15°$; minimizing differences between the own and the recipient's payoff and maximizing the joint payoffs at personal cost), and *altruism* (iSVO $> 57.15°$; maximizing the recipient's payoff at personal cost).

**gSVO.** Additionally, we developed gSVO to assess participants' concern for the ingroup's welfare relative to an outgroup's welfare, without involving any personal stakes, thus ruling out individual-level altruistic preferences as a determinant of choices. Specifically, participants allocate monetary tokens between an unknown ingroup member and an unknown outgroup member. Apart from that, the six items are identical to the standard iSVO measure. Thus, as for iSVO, participants' choices allow for calculation of a continuous gSVO angle, ranging from *strong parochialism* (gSVO $< -12.04°$; maximizing the ingroup member's relative payoff and minimizing the outgroup member's payoff at cost of the ingroup member) to *weak parochialism* ($-12.04° \leq$ gSVO $\leq 22.45°$; maximizing the ingroup member's absolute payoff), *universalism* ($22.45° <$ gSVO $\leq 57.15°$; minimizing the difference between the ingroup and the outgroup member's payoff and maximizing the joint payoffs at cost to the ingroup member), and *xenialism* (maximizing the outgroup member's payoff at the cost of the ingroup member).

**Coin versions.** The coin versions of iSVO and gSVO were used in Study 4 to make the task easier for participants with low numeracy. Fig. S1 shows a sample coin slider: number 1, alternative 2. The coins shown are Ethiopian Birrs. For the coin versions we scaled down amounts from maximally 100 in iSVO/gSVO to maximally 20. Moreover, tokens were shown as coins, removing the extra conversion step from points to monetary payoffs. We allowed payoffs to either be "full" coins (integers) or "halved" coins (digits with one decimal place of .5). Additionally, we reduced the number of options per item from 9 to 7. Note that all our modifications retain the item selectivity of the standard scale, that is, each change in payoff due to a different choice in iSVO and gSVO also results in a payoff change in iSVOcoin and gSVOcoin, respectively. Thus, the ordinal structure of payoffs in the standard and the coin measures has the same monotonic increase (or decrease) between choice options, allowing transformation of responses to the original angle score. Table S1 shows all adapted items.

**Table S1:** *The six (primary) iSVO/gSVO slider measure items in standard and coin versions. Note: Standard version sliders are the same as the Social Value Orientation sliders devised by Murphy* et al. *[S1]. In the iSVO measure, 'self' refers to the payoff of the allocator and 'other' refers to the payoff of an unknown recipient (who can be ingroup or total stranger, i.e., have no marked group membership). In the gSVO measure, 'ingroup' refers to the payoff of an unknown ingroup member and 'outgroup' refers to the payoff of an unknown outgroup member. Choice alternatives were presented in horizontal format in the order in which they appear in the table (from left to right). In the coin version, each payoff value was shown as a picture of multiple coins, e.g., 17 = 17 coins were displayed (Study 1: EUR 0.10 coins; Study 4: ETB 1.00 coins). Also see Figure S1.*

| | | Alternatives, *standard version* | | | | | | | | | Alternatives, *coin version* | | | | | | |
|---|---|---|---|---|---|---|---|---|---|---|---|---|---|---|---|---|---|
| ***Slider*** | | *1* | *2* | *3* | *4* | *5* | *6* | *7* | *8* | *9* | *1* | *2* | *3* | *4* | *5* | *6* | *7* |
| **1** | self / ingroup | 85 | 85 | 85 | 85 | 85 | 85 | 85 | 85 | 85 | 17 | 17 | 17 | 17 | 17 | 17 | 17 |
| | other / outgrp. | 85 | 76 | 68 | 59 | 50 | 41 | 33 | 24 | 15 | 17 | 14.5 | 12.5 | 10 | 7.5 | 5.5 | 3 |
| **2** | self / ingroup | 85 | 87 | 89 | 91 | 93 | 94 | 96 | 98 | 100 | 17 | 17.5 | 18 | 18.5 | 19 | 19.5 | 20 |
| | other / outgrp. | 15 | 19 | 24 | 28 | 33 | 37 | 41 | 46 | 50 | 3 | 4 | 5.5 | 6.5 | 7.5 | 9 | 10 |
| **3** | self / ingroup | 50 | 54 | 59 | 63 | 68 | 72 | 76 | 81 | 85 | 10 | 11 | 12.5 | 13.5 | 14.5 | 16 | 17 |
| | other / outgrp. | 100 | 98 | 96 | 94 | 93 | 91 | 89 | 87 | 85 | 20 | 19.5 | 19 | 18.5 | 18 | 17.5 | 17 |
| **4** | self / ingroup | 50 | 54 | 59 | 63 | 68 | 72 | 76 | 81 | 85 | 10 | 11 | 12.5 | 13.5 | 14.5 | 16 | 17 |
| | other / outgrp. | 100 | 89 | 79 | 68 | 58 | 47 | 36 | 26 | 15 | 20 | 17 | 14.5 | 11.5 | 8.5 | 6 | 3 |
| **5** | self / ingroup | 100 | 94 | 88 | 81 | 75 | 69 | 63 | 56 | 50 | 20 | 18.5 | 16.5 | 15 | 13.5 | 11.5 | 10 |
| | other / outgrp. | 50 | 56 | 63 | 69 | 75 | 81 | 88 | 94 | 100 | 10 | 11.5 | 13.5 | 15 | 16.5 | 18.5 | 20 |
| **6** | self / ingroup | 100 | 98 | 96 | 94 | 93 | 91 | 89 | 87 | 85 | 20 | 19.5 | 19 | 18.5 | 18 | 17.5 | 17 |
| | other / outgrp. | 50 | 54 | 59 | 63 | 68 | 72 | 76 | 81 | 85 | 10 | 11 | 12.5 | 13.5 | 14.5 | 16 | 17 |

### *Data S1: Additional results*

**Manipulation check.** Results supported that individuals identified with their groups (engineering vs. business students) to some extent, as the mean of the ingroup identification scale ($M = 4.65$, $SD = 1.25$) significantly exceeded the scale's midpoint of 4 (one sample t-test: $t(170) = 6.74$, $P < .001$).

**Test-retest reliability.** There was a considerable level of stability in the levels of iSVO and gSVO across measurement occasions ($r(154)_{T1 \to T2} = .66$, 95% CI $[0.56, 0.74]$, $P < 0.001$, for iSVO and $r(154)_{T1 \to T2} = .58$, 95% CI $[0.46, 0.67]$, $P < 0.001$, for gSVO). Likewise, there was a high overlap in the classification of individuals into types based on the theoretically derived cut-offs suggested by [S1]. Specifically, 83.3% of participants were assigned to the same iSVO class and 79.5% of participants were assigned to the same gSVO class across measurement occasions. Moreover, of those 28 participants whose iSVO classification changed, 27 were classified to a neighboring category in the second measurement (e.g., change from 'competitive' to 'individualistic'); only a single participant 'skipped' a category and changed from 'competitive' to 'prosocial.' For gSVO, all 33 participants whose classification changed were classified into a neighboring category in the second measurement (e.g., change from 'strongly parochial' to 'weakly parochial'). We interpret these results as indicating satisfactory levels of test-retest reliability for social preferences over a period of two weeks, both at the individual and the group level [S2–S6].

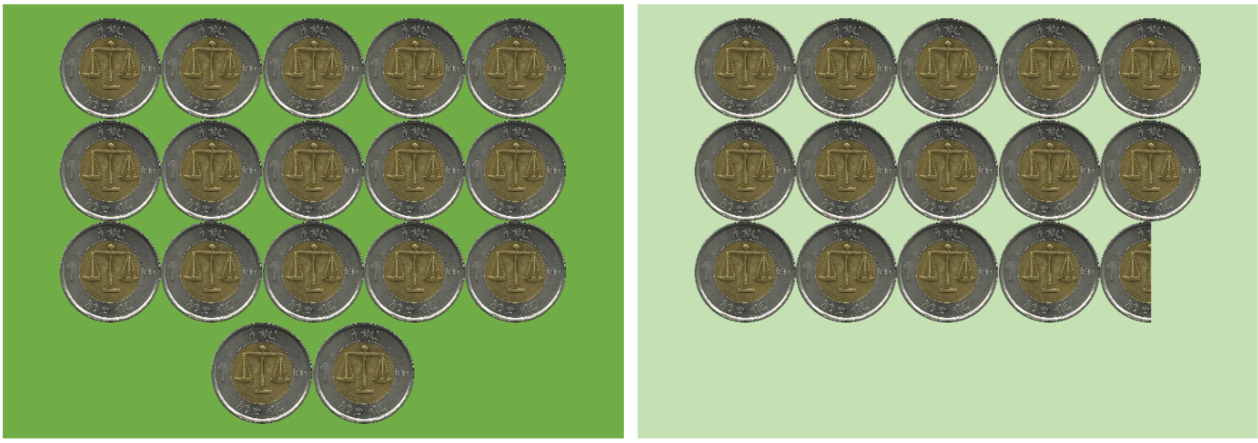

**Figure S1:** *A sample alternative of the coin version slider measure: shown is Slider 1, Alternative 2, i.e., ETB 17 for self/ingroup and ETB 14.5 for other/outgroup. In the coin version, the different sliders were color-coded to facilitate understanding of the task.*

**Convergent and discriminant validity.** Tables S2 and S3 summarize the descriptive statistics of all additional measures assessed in Study 1 as well as their zero-order correlations with gSVO at T1. The tables also indicate our hypotheses about the association between gSVO and the validation measures — based on theoretical considerations or on previous findings on the relation between certain personality traits and individual participation in between-group conflict [S7–S9]. As expected, gSVO was positively correlated with the BGO motivations to maximize equality and joint welfare of both groups (i.e., 'Min diff', 'Max joint ingroup', and 'Max joint outgroup') and negatively correlated with the motivations to maximize the ingroup's absolute or relative payoff ('Max ingroup' and 'Max rel ingroup'; Table S2). There was no association between gSVO and the motivations that mainly focused on maximizing the outgroup welfare without any or even with a negative valuation of the ingroup's welfare (i.e., 'Max outgroup' and 'Max rel outgroup'). Regarding the relations of gSVO with the HEXACO personality dimensions (Table S3), results were likewise in line with expectations. That is, the strongest (negative) association was apparent for honesty-humility ($r(169) = .302$, 95% CI $[0.16, 0.43]$, $P < 0.001$), whereas all other HEXACO dimensions yielded correlations of $r(169) \leq |.155|$. gSVO also showed a negative medium-sized correlation with SDO ($r(169) = -.253$, 95% CI $[-0.39, -0.11]$, $P < 0.001$), as expected. Somewhat surprisingly, however, we found only weak associations of parochialism with GA ($r = -.094$, 95% CI $[-0.24, 0.06]$, $P = 0.22$) and IWAH (on all levels; see Table S3).

Taken together, with only few exceptions, correlations of our novel gSVO measure with other measures were in line with expectations. Correspondingly, there was also no single correlation that was significantly opposite to the expected association. This demonstrates the convergent validity of the gSVO measure in assessing different levels of parochialism. In turn, the size of the correlations (all $r(169)$'s $< |.60|$) corroborate that gSVO represents a unique construct that is not sufficiently captured by any other measure, additionally supporting its discriminant validity.

**Relationship between the standard and the coin-versions of the iSVO and gSVO slider measures.** Finally, we tested the convergence of the iSVO and gSVO standard slider measures (as assessed at T2) with the adapted coin-versions of both measures. iSVO and iSVOcoin showed a strong positive correlation of $r(154) = .83$, 95% CI $[0.78, 0.88]$, $P < .001$. Similarly, gSVO and gSVOcoin showed

**Table S2:** *Zero-order correlations of between-group orientations ('BGO') with gSVO in Study 1. Notes: Based on participants' responses at T1 ($N = 171$). BGO: Between-group orientations measure as devised by Bornstein [S10]. High gSVO values indicate high xenialism.*

| BGO subscale | *M* (*SD*) | Expected relation with gSVO | Correlation with gSVO | *P* | 95% CI of *r* |
|---|---|---|---|---|---|
| Max in-group | 2.23 (3.24) | – | –.573 | <.001 | [–.665, –.462] |
| Max rel in-group | 0.21 (0.87) | – | –.332 | <.001 | [–.460, –.192] |
| Min diff | 2.16 (3.07) | + | .257 | <.001 | [.111, .392] |
| Max out-group | 0.08 (0.33) | + | –.001 | .989 | [–.151, .149] |
| Max rel out-group | 0.01 (0.15) | + | .039 | .612 | [–.112, .188] |
| Max joint in-group | 4.28 (3.24) | + | .257 | <.001 | [.112, .392] |
| Max joint out-group | 0.93 (1.68) | + | .306 | <.001 | [.164, .436] |

**Table S3:** *Zero–order correlations of HEXACO personality factors, SDO, GA, and IWAH with parochialism in Study 1. Note: Based on participants' responses at T1 ($N = 171$). HEXACO personality factors assessed via HEXACO–60 [S11]; SDO: Social dominance orientation measured via scale devised by Cohrs & Asbrock [S12]; GA: Group authoritarianism measured via scale devised by Stellmacher & Petzel [S13]; IWAH: Identification with all humanity measured via scale devised by Macfarlan* et al. *[S14]; gSVO: group–level social value orientation slider measure, high values indicate high xenialism.*

| Measure | *M* (*SD*) | Exp'd relation with gSVO | Obs'd correl. with gSVO | *P* | 95% CI of *r* |
|---|---|---|---|---|---|
| HEXACO | | | | | |
| Honesty–humility | 3.23 (0.69) | + | .302 | < .001 | [.159, .432] |
| Emotionality | 3.03 (0.62) | ? | .049 | .521 | [–.101, .198] |
| Extraversion | 3.41 (0.64) | ? | –.155 | .043 | [–.298, –.005] |
| Agreeableness | 3.15 (0.52) | ? | .104 | .177 | [–.047, .250] |
| Conscientiousness | 3.54 (0.56) | ? | –.074 | .335 | [–.222, .077] |
| Openness to experience | 3.26 (0.63) | ? | .047 | .539 | [–.104, .196] |
| SDO | 3.35 (0.69) | – | –.253 | < .001 | [–.388, –.107] |
| GA | 3.27 (0.75) | – | –.094 | .220 | [–.241, .057] |
| IWAH | | | | | |
| Personal level | 4.45 (0.44) | ? | –.093 | .226 | [–.240, .058] |
| Group level | 3.17 (0.58) | – | –.105 | .172 | [–.251, .046] |
| Collective level | 3.00 (0.65) | + | .049 | .552 | [–.102, .198] |

a strong positive correlation of $r(154) = .77$, 95% CI $[0.70, 0.83]$, $P < .001$. These findings indicate that the adapted coin-versions are well-suited as alternative measures of individual- and group-level social preferences in populations characterized by low numeracy.

## S2 Supplementary information and results for Study 2

### *Methods S2: The IPD game*

Participants' in Study 2 played the Intergroup Prisoner's Dilemma (IPD) game [S10]. The game is played by an even number of $N$ players $i$, who are each assigned to one of two groups. Groups are equal in size, that is, each group has $n = N/2$ players, with $I \in \{1, \ldots, n\}$ denoting the set of players in the ingroup and $O = \{n+1, \ldots, N\}$ denoting the set of players in the outgroup. Each player is endowed with $e$ tokens and decides in private how many tokens, $g_i$, to contribute to a between-group conflict pool (with $0 \leq g_i \leq e$). Tokens not contributed are retained privately.

Table S4 shows the payoff changes for all players per token kept vs. contributed to the between-group conflict pool. Contributions to the between-group conflict pool are multiplied by a constant $k$ (with $1 < k < n$) and are equally distributed among all ingroup players. Thus, each ingroup player receives $(k \cdot g_i)/n$ given a player's contribution $g_i$. In addition, contributions to the between-group conflict pool are multiplied by a constant $k$ and reduce the payoff of each outgroup player equally by the amount $(k \cdot g_i)/n$ given a player's contribution $g_i$. Tokens not contributed are directly transferred to the private account of the player (without affecting the payoffs of the other ingroup and the outgroup members). The resulting individual payoff function is:

$$\pi_i = e - g_i + \frac{k}{n} \sum_{j=1}^{n} g_j - \frac{k}{n} \sum_{h=1}^{N} g_h.$$

In our experiment, we set the parameters as follows: $N = 6$ (i.e., each group had the size $n = 3$), $e = 10$, with each token worth EUR 1.00, and $k = 1.5$. Thus, for each token contributed to the between-group conflict pool the payoff of the contributing player and each ingroup member increased by EUR 0.50 and the payoff of each outgroup member decreased by EUR 0.50.

By implication, keeping all tokens constitutes the dominant (selfish-rational) option, maximizing the player's payoff, regardless of what the other players do. Contributing all tokens to the between-group conflict pool maximizes the ingroup's aggregated payoff and minimizes the outgroup's aggregated payoff. Note that contributions to the between-group conflict pool are collectively destructive. That is, if all players contribute to the between-group conflict pool, they are all worse off compared to when they all keep their endowments. This mirrors the structure of between-group conflicts.

### *Data S2: Additional results*

**Manipulation check.** Results supported that individuals highly identified themselves with their supporter group, as the mean of the ingroup identification scale ($M = 5.21$, $SD = 1.14$) significantly exceeded the scale's midpoint of 4 (two-sided one sample t-test: $t(192) = 14.75$, $P < .001$).

**Behavior in the IPD game.** The average contribution to the between-group conflict pool in the IPD was $M = 4.13$ ($SD = 3.56$) out of 10 tokens. In fact, 71% ($n = 137$) of the participants contributed

**Table S4:** *Behavioral options and payoff conseuqences in the games used in Studies 2 and 3. IPD: 'Intergroup Prisoner's Dilemma' as devised by Bornstein [S10]. IPD-MD: 'Intergroup Prisoner's Dilemma – Maximizing Differences' as devised by Halevy* et al. *[S15]. Marginal payoffs 'X / Y / Z' indicate the changes in payoffs for the respective player (X), each of the contributor' ingroup members (Y), and each of the outgroup members (Z) when the player invests 1 token into the respective behavioral option. 'n/a': Behavioral option not available in that game.*

| **Game** | *Behavioral option* | | |
|---|---|---|---|
| | *Keep* | *Between-group pool* | *Within-group pool* |
| ***IPD*** (Study 2) | $+1/\pm0/\pm0$ | $+0.50/+0.50/-0.50$ | n/a |
| ***IPD-MD*** (Study 3) | $+1/\pm0/\pm0$ | $+0.50/+0.50/-0.50$ | $+0.50/+0.50/\pm0$ |

at least some tokens to the between-group conflict pool. We conducted linear regression analyses to investigate how participants' level of both iSVO and gSVO affected their contribution behavior. Results are shown in Table S5 and discussed below together with those of Study 3.

## S3 Supplementary information and results for Study 3

### *Methods S3: The IPD-MD game*

Participants played the 'Intergroup Prisoner's Dilemma – Maximizing Difference' game devised by Halevy *et al.* [S15]. The IPD-MD game is played by $N$ players $i$, who are assigned to two different groups. Groups are equal in size, that is, each group has $n = N/2$ players, with $I \in \{1, \ldots, n\}$ denoting the set of players in the ingroup and $O = \{n+1, \ldots, N\}$ denoting the set of players in the outgroup. Each player is endowed with $e$ tokens and decides in private how many tokens, $g_i$, to contribute to a between-group conflict pool (with $0 \leq g_i \leq e$) and how many tokens, $h_i$, to contribute to a within-group cooperation pool (with $0 \leq h_i \leq e$, and $g_i + h_i \leq e$). Tokens not contributed are kept privately.

Table S4 shows the payoff changes for all players per token kept versus contributed to the between-group conflict pool versus contributed to the within-group cooperation pool. Contributions to the between-group conflict pool are multiplied by a constant $k$ (with $1 < k < n$) and are equally distributed among all ingroup players. Thus, each ingroup player receives $(k \cdot g_i)/n$ from a player's contribution $g_i$. In addition, contributions to the between-group conflict pool are multiplied by a constant $k$ and reduce the payoff of each outgroup player equally by the amount $(k \cdot g_i)/n$ from a player's contribution $g_i$. Similarly, contributions to the within-group cooperation pool are multiplied by the constant $k$ and are equally distributed among all ingroup players. Thus, each ingroup player receives $(k \cdot h_i)/n$ from a player's contribution $h_i$, too. In contrast to the between-group conflict pool, however, contributions to the within-group cooperation pool have no (negative) effect on the outgroup members' payoffs. Tokens not contributed to any pool are directly transferred to the individual account of the player (without affecting the payoff of any other player). The resulting individual payoff function is:

$$\pi_i = e - g_i - h_i + \frac{k}{n}\sum_{j=1}^{n}(g_j + h_j) - \frac{k}{n}\sum_{h=1}^{N} g_h.$$

In our experiment, we set the parameters as follows: $N = 6$ (i.e., each group had the size $n = 3$), $e = 10$, with each token worth USD 1.00, and $k = 1.5$. Thus, for each token contributed to the

between-group conflict pool or to the within-group cooperation pool, the payoff of the contributing player and each ingroup member increased by USD 0.50; the payoff of each outgroup member decreased by USD 0.50 for each token contributed to the between-group conflict pool.

As in the IPD, keeping all tokens in the IPD-MD constitutes the dominant (selfish-rational) strategy, maximizing the player's payoff, regardless of what the other players do. Both contributions to the between-group conflict pool and contributions to the within-group cooperation pool maximize the ingroup's aggregated payoff. Thus, players are indifferent between contributions to these pools if they simply want to maximize the ingroup's welfare without considering the outgroup's welfare. However, contributions to the between-group conflict pool minimize the outgroup's aggregated payoff (indicating between-group aggression or competition), whereas contributions to the within-group conflict pool maximize collective welfare.

### *Data S3: Additional results*

**Manipulation check.** Results supported that individuals highly identified with their political group, as the mean of the ingroup identification scale ($M = 5.15$, $SD = 1.18$) significantly exceeded the scale's midpoint of 4 (two-sided one sample t-test: $t(424) = 20.07$, $P < .001$).

**Robustness test: ingroup vs. unknown/'stranger' recipient in the iSVO.** There was no significant difference in the level of iSVO when the recipient was a neutral partner vs. an ingroup member (independent samples t-test: $t(423) = 0.85, P = .396$, $d = 0.08$). This is in line with previous research showing that the level of prosociality toward unknown others is similar to the level of prosociality toward ingroup members [S16, S17]. We therefore pooled the two conditions for further data analyses.

**Behavior in the IPD-MD game.** The average contribution to the between-group conflict pool in the IPD-MD was $M = 1.39$ ($SD = 2.09$) out of 10 tokens and the average contribution to the within-group cooperation pool was $M = 3.36$ ($SD = 3.17$). As expected, contributions to the between-group conflict pool were much lower than in the IPD (Study 2) where participants had to engage in between-group conflict to benefit their ingroup. Still, 46% ($n = 195$) of participants contributed at least some tokens to the between-group conflict pool; to the within-group pool 71% ($n = 300$) participants contributed at least some tokens.

The regression results in Table S5 show that both iSVO (positively) and gSVO (negatively) contribute to predicting *between-group pool* contributions in Study Study 2, Models (1)-(3), and Study 3, Models (4)-(6). No significant interaction between the two measures in predicting *between-group pool* contributions is observed in these models. In contrast, iSVO (positive) and gSVO (positive) both significantly contribute to explaining *within-group pool* contributions in Study 3, Models (7)-(9). Here, Models (8) and (9) also identify a significant (positive) interaction of the two measures. As can be seen from Figure 2, this interaction is such that gSVO positively predicts contributions at high levels of iSVO, while this relation flattens out, turning even slightly negative, for low levels of iSVO. All results hold when additionally controlling for participants' age and gender, Models (3), (6), and (9).

**Theoretical prediction of behavioral separation in the IPD/IPD-MD game.** Assume agents' preferences are described by

$$u(x, x_i, x_o) = x + \alpha \cdot x_i + \gamma \cdot x_o, \qquad \text{(eq. 1)}$$

**Table S5:** *Results of OLS regressions for Studies 2 and 3. The dependent variables are contributions (possible range = 0 - 10) to the respective pools in the IPD game (Study 2) or IPD-MD game (Study 3). Noteworthy observations: (i) iSVO and gSVO independently predict contributions to the between-group pool across studies, i.e., behavior that is (financially) harmful to the outgroup; (ii) for contributions to the within-group pool iSVO and gSVO additionally show a significant interaction. Both results are robust to controlling for age and participants' identification as 'male' (yes/no).*

| | *Dependent variables:* | | | | | | | | |
|---|---|---|---|---|---|---|---|---|---|
| | Study 2 | | | Study 3 | | | | | |
| | Between-group pool | | | Between-group pool | | | Within-group pool | | |
| | (1) | (2) | (3) | (4) | (5) | (6) | (7) | (8) | (9) |
| iSVO | $0.078^{***}$ | $0.081^{***}$ | $0.078^{***}$ | $0.021^{**}$ | $0.026^{**}$ | $0.021^{**}$ | $0.079^{***}$ | $0.060^{***}$ | $0.060^{***}$ |
| | (0.018) | (0.019) | (0.018) | (0.008) | (0.009) | (0.008) | (0.011) | (0.012) | (0.012) |
| gSVO | $-0.061^{***}$ | $-0.049^{+}$ | $-0.059^{***}$ | $-0.028^{***}$ | $-0.015$ | $-0.028^{***}$ | $0.024^{*}$ | $-0.029^{+}$ | $-0.029^{+}$ |
| | (0.015) | (0.027) | (0.015) | (0.007) | (0.012) | (0.007) | (0.009) | (0.016) | (0.016) |
| iSVO × gSVO | | $-0.0005$ | | | $-0.0005$ | | | $0.002^{***}$ | $0.002^{***}$ |
| | | (0.001) | | | (0.0004) | | | (0.0005) | (0.0005) |
| Age | | | 0.043 | | | $-0.014$ | | | 0.003 |
| | | | (0.038) | | | (0.010) | | | (0.013) |
| Male | | | 0.304 | | | 0.053 | | | 0.028 |
| | | | (0.582) | | | (0.204) | | | (0.276) |
| Constant | $3.226^{***}$ | $3.195^{***}$ | 1.807 | $1.349^{***}$ | $1.286^{***}$ | $1.742^{***}$ | $0.905^{***}$ | $1.165^{***}$ | $1.048^{+}$ |
| | (0.424) | (0.429) | (1.190) | (0.197) | (0.202) | (0.397) | (0.270) | (0.273) | (0.538) |
| Observations | 193 | 193 | 193 | 425 | 425 | 425 | 425 | 425 | 425 |
| $R^2$ | 0.114 | 0.115 | 0.122 | 0.040 | 0.044 | 0.045 | 0.214 | 0.243 | 0.243 |
| Adjusted $R^2$ | 0.105 | 0.101 | 0.103 | 0.035 | 0.037 | 0.035 | 0.210 | 0.238 | 0.234 |
| Resid. Std. Error | 3.369 | 3.376 | 3.373 | 2.052 | 2.050 | 2.052 | 2.813 | 2.764 | 2.770 |
| F Statistic | $12.217^{***}$ | $8.212^{***}$ | $6.505^{***}$ | $8.704^{***}$ | $6.393^{***}$ | $4.892^{***}$ | $57.391^{***}$ | $45.073^{***}$ | $26.932^{***}$ |

*Note:* $^{+}$p<0.10; $^{*}$p<0.05; $^{**}$p<0.01; $^{***}$p<0.001

as explained in the main paper. For simplicity, also assume that, in case multiple ingroup and/or outgroup members are involved in the respective interaction, their payoffs are simply summed. Then, with mild abuse of notation, the marginal utility of allocating one more unit to pool B, i.e., the between-group pool, in the IPD and the IPD-MD is given by

$$u'(-\frac{1}{2}, 2\times+\frac{1}{2}, 3\times-\frac{1}{2}) = -\frac{1}{2} + \alpha - \frac{3}{2}\gamma$$

and is thus positive when $\gamma < \frac{(2\alpha-1)}{3}$. Moreover, the marginal utility of one more unit in pool A, i.e., the within-group pool, in the IPD is: $u'(-\frac{1}{2}, 2\times+\frac{1}{2}, 0) = -\frac{1}{2} + \alpha$ which is positive for $\alpha > \frac{1}{2}$. Moreover, investing in A is preferred to investing in B when $\gamma > 0$. Figure S2 marks the areas of the $(\alpha, \gamma)$-space for which these three conditions hold.

Predicted behavior in the games can be inferred as follows. *IPD:* Agents who prefer investing via pool B (i.e., players whose preferences fall into the '$B \succ P$'-area in Fig. S2) should choose the within-group pool and everyone else should invest via P, i.e., keep their endowment. *IPD-MD:* Here, a

third investment option, pool A, is added, so players now compare the marginal benefits of investing via A, B, and P. This 'activates' additional players who now contribute via A instead of keeping via P: those for whom $A \succ P$ but not $A \succ B$. Moreover, introducing pool A makes those players 'switch' from pool B to pool A, namely those for whom $A \succ B$ and $B \succ P$, i.e., players with preferences in the highlighted triangle in Fig. S2. Intuitively, these are players who are sufficiently prosocial, $\alpha > 1/2$, not spiteful toward the outgroup, and moderately ingroup-favoring, $0 < \gamma < (2\alpha - 1)/3$.

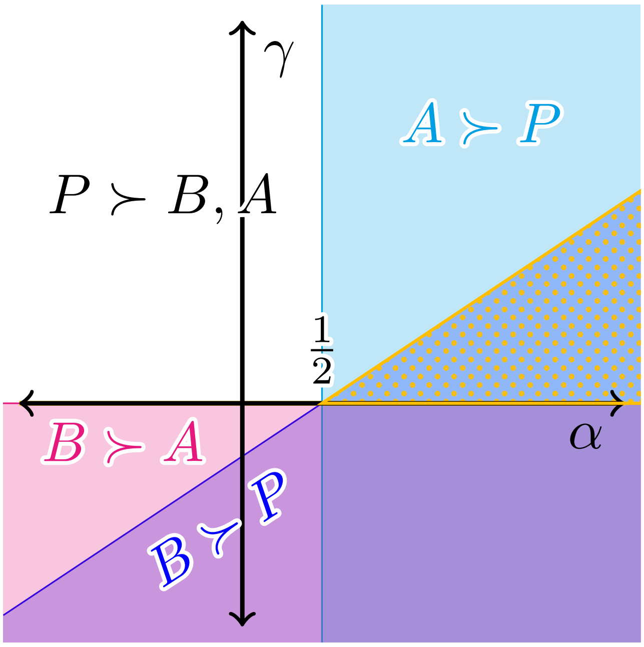


**Figure S2:** *Investment preferences (coloring) for a marginal unit in the IPD and IPD-MD games given preferences $(\alpha, \gamma)$. 'X $\succ$ Y' means investment in pool X is preferred to investment in pool Y; pools: P = 'Private', B = 'Between-group', A = 'Within-group'. Individuals with preferences in the dark shaded triangle are those who are predicted to switch from investments via pool B in the IPD to investments via pool A in the IPD-MD.*

## S4 Supplementary information and analysis for Study 4

### *Data S4: Moderation analysis*

Table S6 shows the results of our logistic regressions of self-reported involvement in violent conflict in the last six months (binary variable: yes/no) on iSVO, gSVO, their interaction, and participant age (as estimated by the field researcher, LG). Results show a significant and robust interaction of iSVO and gSVO in predicting conflict involvement. Figure S3 visualizes the predicted association for two levels of gSVO: the $20^{th}$ and the $80^{th}$ percentile of the $N = 111$ participants' observed gSVO scores. The interaction result thus implies that participants at high levels of gSVO, i.e., the relatively more universalistic, show a lower change in iSVO when exposed to conflict compared to those at low levels of gSVO, i.e., the relatively more parochialistic participants.

## S5 Supplementary information and results for Study 5

### *Data S5: Additional results*

**Robustness test.** We only report tests for zero-order level differences in iSVO and gSVO in the main text. Our preregistered analysis strategy for Study 5 were respective ANOVAs, though. Results

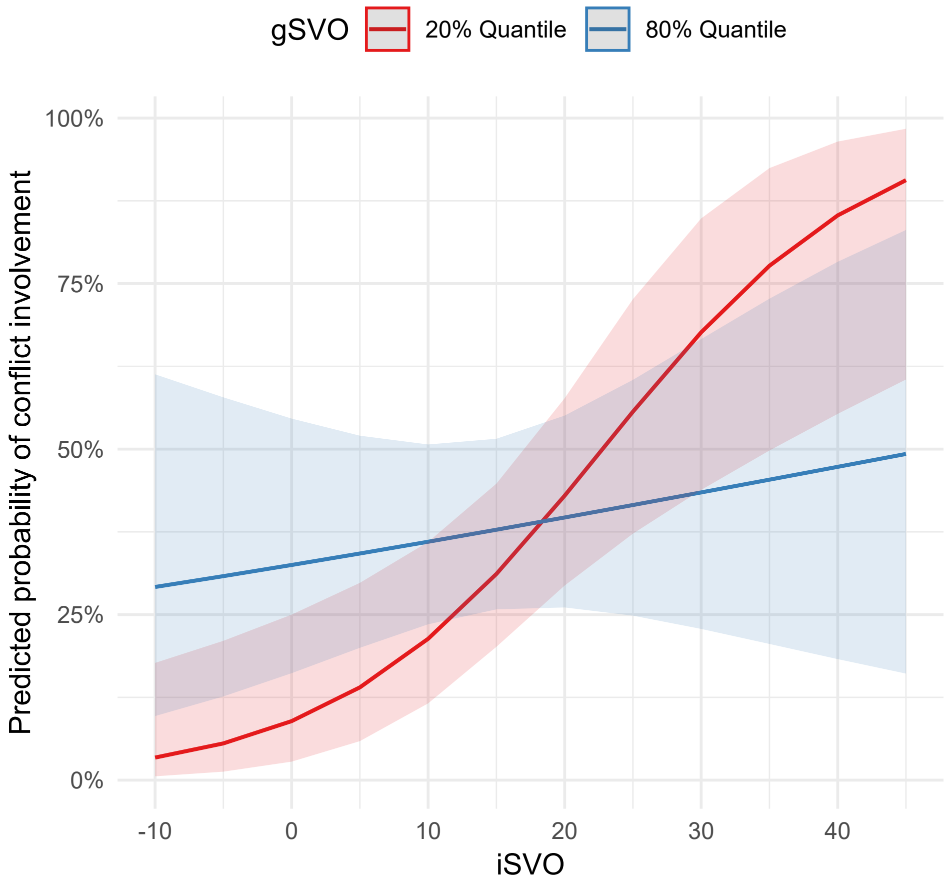


**Figure S3:** *Predicted association of iSVO with likelihood of self-reported conflict involvement in the six months prior to day of data collection for different levels of gSVO ($20^{th}$ and $80^{th}$ quantile), controlling for approximate age of participant; confidence bands indicate 95% CIs.*

are robust when using the preregistered method. Our replication materials include the respective calculations.

**Within-participant analysis.** In addition to manipulating perceived conflict intensity between groups, we leveraged the within-participants design of this study to simultaneously test for effects of varying subjective identification strengths with different ingroups on iSVO and gSVO. Participants in the 'identification' condition ($N = 236$) completed the iSVO and gSVO measures twice: once for the group pairing containing the ingroup which they ranked as most important for their self-conceptualization and once for the pair with the least important ingroup. Results showed a significant effect of this manipulation on gSVO ($M_{high} = 18.00$ vs. $M_{low} = 22.56$, $t = 2.47$, $P = 0.01$, $d = 0.25$), but not on iSVO ($M_{high} = 25.60$ vs. $M_{low} = 25.38$, $t = 0.15$, $P = 0.88$, $d = 0.02$).

**Table S6:** *Logistic regression analyses testing the association of reported conflict involvement in the previous six months with gSVO and iSVO and their interaction in Study 4.*

| | *Dependent variable:* | | | |
|---|---|---|---|---|
| | Recent conflict involvement (yes/no) | | | |
| | (1) | (2) | (3) | (4) |
| iSVO | $0.048^{*}$ | $0.047^{*}$ | $0.070^{**}$ | $0.070^{**}$ |
| | (0.021) | (0.021) | (0.024) | (0.024) |
| gSVO | 0.004 | 0.004 | $0.074^{*}$ | $0.076^{*}$ |
| | (0.017) | (0.017) | (0.034) | (0.034) |
| iSVO × gSVO | | | $-0.004^{*}$ | $-0.004^{*}$ |
| | | | (0.002) | (0.002) |
| Approx. age | | 0.009 | | 0.017 |
| | | (0.028) | | (0.029) |
| Constant | $-1.298^{***}$ | $-1.529^{+}$ | $-1.696^{***}$ | $-2.139^{*}$ |
| | (0.392) | (0.801) | (0.451) | (0.882) |
| Observations | 111 | 111 | 111 | 111 |
| Log Likelihood | −70.208 | −70.153 | −67.062 | −66.887 |
| Akaike Inf. Crit. | 146.416 | 148.306 | 142.125 | 143.774 |
| *Note:* | $^{+}p<0.10$; $^{*}p<0.05$; $^{**}p<0.01$; $^{***}p<0.001$ | | | |

# Supplementary References


S1. Murphy, R. O., Ackermann, K. A. & Handgraaf, M. J. J. Measuring Social Value Orientation. *Judgment and Decision Making* **6,** 771–781 (2011).

S2. Dasgupta, U., Gangadharan, L., Maitra, P. & Mani, S. Searching for preference stability in a state dependent world. *Journal of Economic Psychology* **62,** 17–32. ISSN: 01674870 (2017).

S3. Volk, S., Thöni, C. & Ruigrok, W. Temporal stability and psychological foundations of cooperation preferences. *Journal of Economic Behavior and Organization.* ISSN: 01672681 (2011).

S4. Carlsson, F., Johansson-Stenman, O. & Nam, P. K. Social preferences are stable over long periods of time. *Journal of Public Economics* **117,** 104–114. ISSN: 0047-2727 (2014).

S5. Greiff, M., Ackermann, K. A. & Murphy, R. O. Playing a Game or Making a Decision? Methodological Issues in the Measurement of Distributional Preferences. *Games* **9,** 80 (2018).

S6. O'Brien, B. & Viramontes, J. L. Willingness to pay: a valid and reliable measure of health state preference? *Medical decision making : an international journal of the Society for Medical Decision Making* **14,** 289–297. ISSN: 0272-989X (1994).

S7. Aaldering, H. & Böhm, R. Parochial Versus Universal Cooperation: Introducing a Novel Economic Game of Within- and Between-Group Interaction. *Social Psychological and Personality Science* **11,** 36–45. ISSN: 1948-5506 (2020).

S8. Thielmann, I. & Böhm, R. Who Does (Not) Participate in Intergroup Conflict? *Social Psychological and Personality Science* **7,** 778–787. ISSN: 1948-5506 (2016).

S9. Halali, E., Dorfman, A., Jun, S. & Halevy, N. More for Us or More for Me? Social Dominance as Parochial Egoism. *Social Psychological and Personality Science* **9,** 254–262. ISSN: 1948-5506 (2018).

S10. Bornstein, G. The free-rider problem in intergroup conflicts over step-level and continuous public goods. *Journal of Personality and Social Psychology* **62,** 597–606. ISSN: 1939-1315 (1992).

S11. Ashton, M. C. & Lee, K. The HEXACO-60: a short measure of the major dimensions of personality. *Journal of Personality Assessment* **91,** 340–345. ISSN: 0022-3891 (2009).

S12. Cohrs, J. C. & Asbrock, F. Right–wing authoritarianism, social dominance orientation and prejudice against threatening and competitive ethnic groups. *European Journal of Social Psychology* **39,** 270–289. ISSN: 0046-2772 (2009).

S13. Stellmacher, J. & Petzel, T. Authoritarianism as a Group Phenomenon. *Political Psychology* **26,** 245–274. ISSN: 0162895X (2005).

S14. Macfarlan, S. J., Walker, R. S., Flinn, M. V. & Chagnon, N. A. Lethal coalitionary aggression and long-term alliance formation among Yanomamö men. *Proceedings of the National Academy of Sciences of the United States of America* **111,** 16662–16669. ISSN: 0027-8424 (2014).

S15. Halevy, N., Bornstein, G. & Sagiv, L. "In-Group Love" and "Out-Group Hate" as Motives for Individual Participation in Intergroup Conflict: A New Game Paradigm. *Psychological Science* **19,** 405–411. ISSN: 09567976 (2008).

S16. Böhm, R., Fleiß, J. & Rybnicek, R. On the Stability of Social Preferences in Inter-Group Conflict: A Lab-in-the-Field Panel Study. *Journal of Conflict Resolution* **65,** 1215–1248. ISSN: 0022-0027 (2021).

S17. Rahal, R.-M., Fiedler, S. & de Dreu, C. K. W. Prosocial Preferences Condition Decision Effort and Ingroup Biased Generosity in Intergroup Decision-Making. *Scientific Reports* **10,** 10132. ISSN: 2045-2322 (2020).
